# Spatiotemporal evolution of urban populations and housing: A dynamic utility-driven market-mediated model

**DOI:** 10.1371/journal.pone.0282583

**Published:** 2023-04-07

**Authors:** P. A. Robinson, A. McInnes, Somwrita Sarkar

**Affiliations:** 1 School of Physics, University of Sydney, Sydney, New South Wales, Australia; 2 School of Architecture, Design, and Planning, The University of Sydney, Sydney, New South Wales, Australia; Institute for Advanced Sustainability Studies, GERMANY

## Abstract

A model of the spatiotemporal evolution of urban areas is developed that simultaneously includes the effects on household utility of geography, population density, income distribution, and household preference for characteristics of dwellings and neighbors. The result is a utility function whose structure is similar to that of the energy of interacting spin systems in external fields. Spatiotemporal housing market evolution then results via transactions driven by increases in utility and changes in numbers of households and dwellings. It is shown that the model successfully predicts formation of monocentric and polycentric urban areas, stratification by wealth, segregation due to preferences for housing or neighbors, and the balance of supply and demand. These results go well beyond those of prior models that each dealt with subsets of these phenomena, and do so within a single, unified framework. Potential generalizations are discussed and further applications are suggested.

## 1 Introduction

The world is undergoing accelerating urbanization, with the projection that by 2050 approximately 68% of its 9.5 billion population will live in urban areas [1]. Therefore, it is critical to understand the mechanisms of formation and evolution of cities and towns, and how the population is distributed within them.

Urban structure is dynamic: people continually move as a result of changing pressures of affordability, employment, and other factors that affect housing preference, including location, thereby altering urban structure. In turn, the existing structure affects the opportunities that people have given their choices through such influences as congestion and accessibility of employment, education, recreation, health services, and other social infrastructure [2]. For example, the Equality of Opportunity project studies very large data sets of families’ movements across the US over generations, finding that urban structures play a large role in social and economic outcomes, alongside income and demographics [3, 4]. These studies show that urban structure is tied to the socioeconomic dynamics of choices exercised by individual agents within the market. Therefore, a model that takes into account such a longitudinal evolution of urban structure, driven by individual choices, market forces, and government interventions, would be desirable.

The formation, structure, and evolution of cities has been studied for nearly two centuries [5–9]. Cities can be viewed as complex systems that are shaped by economic and social factors under spatial and geographical constraints [7, 10, 11], resulting in agglomerations of people, housing, infrastructure, and organizations such as businesses, with associated consequences for economic, social, and transport interactions. Resulting urban models have been based on principles drawn from as far afield as economic geography [5], economics [7, 10], physics [6, 12], synergetics [13], complex systems [5, 9, 14, 15], and network science [6, 9]. These have attempted to answer questions such as how cities form initially via agglomeration, how land uses within them organize in response to economic and functional organization of activity, whether or not different types of activities and people are segregated, effects of income, transport, and other economic and technological factors, and the role of geographical constraints.

Von-Thunen’s 1826 model of agricultural land use explained how rent and transport cost trade-offs result in land uses organizing in concentric circles around a market town, based on the relative price of land and profitability of different activities [5, 8]. This model led to the development of monocentric and polycentric city models [7, 10] but these do not explain how a town center or central business district (CBD) arises in the first place. Christaller’s Central Place Theory [16] involved a hierarchical arrangement with successively smaller market towns organized around larger ones on a hexagonal grid. But this was a purely descriptive model that did not explain why such an arrangement would arise.

Schelling [5, 11] addressed social, rather than economic, aspects of the population distribution within cities. His agent-based model addressed the question of segregation or integration of different social groups on the basis that individuals have a slightly higher preference for having similar individuals as neughbors. His main finding was that starting from even a completely balanced and integrated system, where everyone’s preferences are satisfied, even a single individual swap can trigger a cascade of local swaps in the system, where individuals adjust their locations according to their only very slightly higher preference for similar neighbors. In other words, an integrated solution is an unstable equilibrium which reorganizes globally in response to even small local changes. The end state is segregated into large clusters of similar individuals, separated by sharp boundaries from other such clusters. He thus found that segregation can occur if people move to satisfy slight preferences for similarity, even if they would have been satisfied with a totally integrated spatial arrangement and only wished not to be an isolated member of their group. The model has since been extensively applied to neighborhood social and residential dynamics and their relationships to urban structure [17], physical clustering [18], and economic interactions [19]. In a related vein, Axelrod and others studied the evolution of individual views and preferences, influenced both by views that are common across the whole society and ones that are local; he found that fragmentation into subgroups is possible if the influence of general views is not strong enough [20–23].

Most economic and social models have considered only simple approximations to the spatial structure of a city—mostly with linear, circularly symmetric, or grid-based geometries [5–8, 10, 11, 20, 21, 24]. However, cities are typically more irregular in shape, must obey geographical constraints, and the question of how they form and grow must be considered. More recent models have thus focused more on the spatial structure of cities, often using physical analogs to describe or explain more realistic features. For example, fractal geometry has been used to describe multiscale city morphology, sometimes in connection with scaling theories of economic and social attributes such as of mean income vs. city size [6, 25]. Consequently, physical processes that produce fractal structures in other contexts have been used to try to reproduce urban morphologies. These include city formation via diffusion-limited-aggregation (DLA) of immigrants to a city, sometimes with modified probabilities of aggregation, preferential attachment, or directed percolation [12, 26]; models of economically driven migration [13]; self-organization that can result in fractal filling of space [14]; agent-based models based on agglomeration principles such preferential attachment [6, 9, 15]; or network models that take into account the structure of transport networks in conjunction with urban structure [6, 9]. Finally, transport models consider the joint spatial structure of economic and social aspects in parallel with the structure of the transport networks that support spatial interactions [6]. These include flows of people, goods, and ideas at timescales from daily commuting and deliveries up to internal long term migration due to factors such as employment relationships and housing needs [6]. Thus, while the principal aim of many urban models has been to explain why and how urban structure takes the form it does, transportation models’ central aim is to estimate flows of people within and between cities.

Models such as those mentioned above each focus on a small set of urban features or processes, while leaving many others unaccounted for. While it is not feasible to incorporate all conceivable aspects in one step of model development, here we construct a unified model that brings together numerous previously disparate aspects within a single framework that includes spatial and geographical effects, household dwelling preferences and utility, dwelling characteristics, income constraints, interactions between people, and market transactions. No claim is made that this is exhaustive, and limitations are discussed below, but we show that a few basic assumptions give rise to a wide range of economic, social, and physical-geographic effects that evolve over time. These include agglomeration into a hierarchy of cities and towns, monocentric versus polycentric growth, income-based population stratification, and social segregation—a variety of outcomes that has not previously emerged from a single model, let alone a mechanistic one. Further extensions are outlined to address a wider range of problems in the future, notably to extend the model to include transport and investment.

In Sec. 2 we introduce our description of household characteristics and housing preferences, including price sensitivity based on household income. Section 3 describes a resulting utility function that includes household-independent contributions as well as components due to housing preference and interactions with other households. Price sensitivity and income are discussed in more detail in Sec. 4 and the way in which the resulting utility determines housing prices is covered in Sec. 5. Evolution of supply, demand, preferences, and characteristics of households and housing is discussed in Sec. 6, including market entry and exit of households and dwellings. The numerical approach to market simulation is outlined in Sec. 7 and simulations are presented in Sec. 8 to illustrate the core components of the model and verify that its dynamics reproduce key observed features of urban structure and housing market dynamics, including city formation, wealth stratification, agglomeration, segregation, and the effects of supply and demand. Section 9 summarizes the main outcomes and suggests further directions for future application and generalization.

## 2 Household preference and characteristic vectors

We suppose that there are *N* households, each of which is labeled with an index *p* = 1, …, *N* and is distinguished by the spatial location of their dwelling **r**(*p*), which can designate an apartment or even a room if necessary when different households occupy different parts of a structure; no two households can have the same **r** at the same time. In our numerical examples, we treat **r** as two-dimensional, but a third coordinate can be used in multistorey buildings.

We suppose that each household *p* is characterized by certain demographic attributes such as number and age of members, education, race, and religion which other households might take into account, fairly or not, in their preferences for neighbors. These are summarized in a dimensionless (*m*+ 1)-element vector **q**(*p*) with

$$\begin{array}{c} q\left(p\right)=[q_{0}\left(p\right),q_{1}\left(p\right),\ldots ,q_{m}\left(p\right)], \end{array}$$ (1)

where *m* may well be large. In the present version of the model, we set *q*_0_ = 1 for all households, merely indicating the presence of the household, which factors into issues such as economies of scale when population concentrations occur; alternatively it could be set equal to the number of household members, wealth, or some other proxy for the household’s overall production and consumption of services; i.e., a measure of household socioeconomic influence.

Households also have housing preferences denoted by a dimensionless (*n* + 1)-element unit vector

$$\begin{array}{c} s\left(p\right)=[s_{0}\left(p\right),s_{1}\left(p\right),\ldots ,s_{n}\left(p\right)], \end{array}$$ (2)

where *n* is likely to be large. Preferences can include such as wanting to live in a certain neighborhood or area (e.g., close to mountains or the sea), or close to employment, public transport, schools, or similar neighbors. For convenience in much of the analysis, we also define

$$\begin{array}{c} s^{\prime }\left(p\right)=\left[s_{1}\left(p\right),\ldots ,s_{n}\left(p\right)\right]. \end{array}$$ (3)

**Fig 1:**
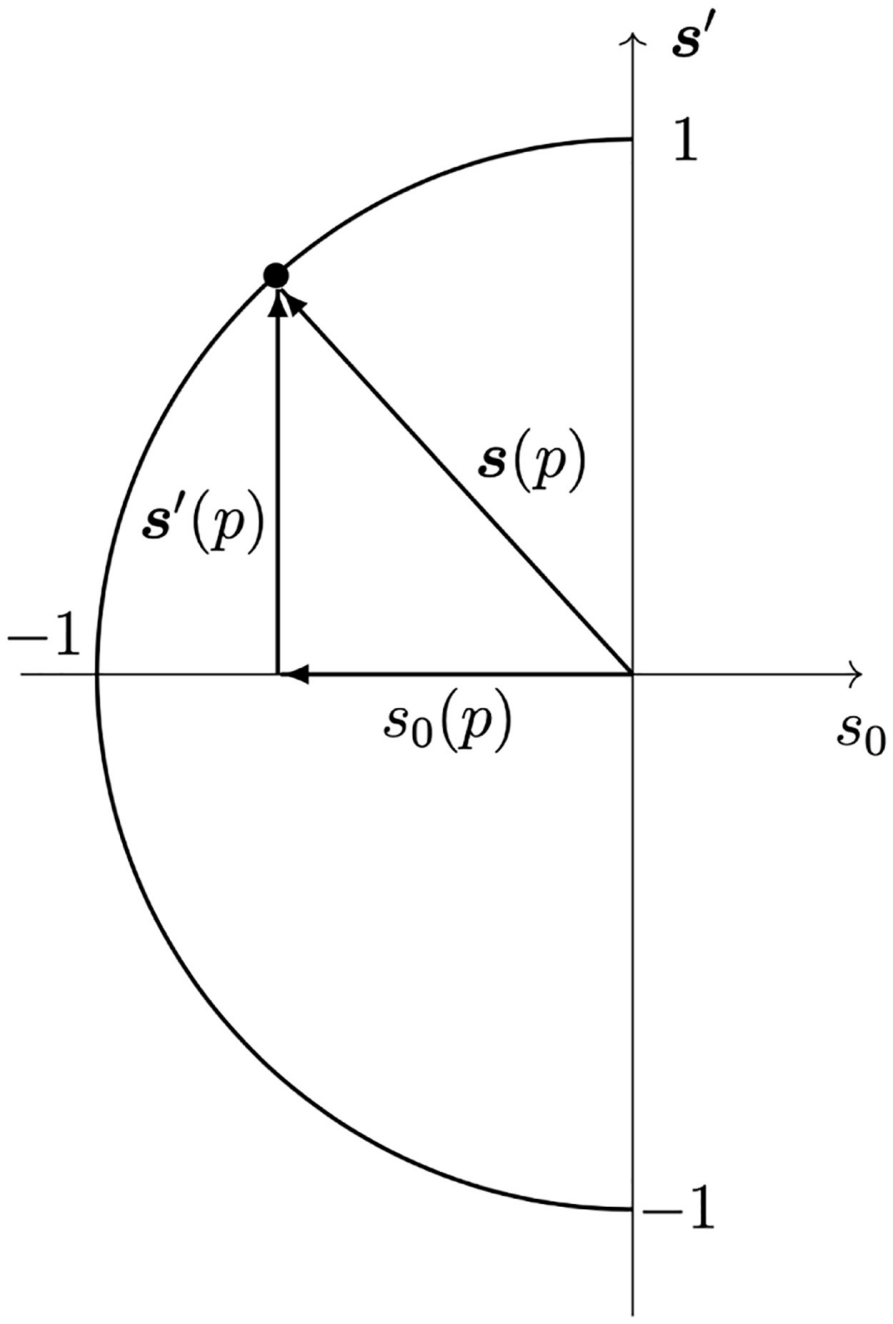
Schematic of the unit preference vector s(*p*) of household *p*, its component *s*_0_(*p*) and the multidimensional remainder vector s′(*p*). As *s*_0_(*p*) approaches −1 wealth decreases and the magnitude of remaining preferences |**s**′(*p*)| is reduced accordingly; the richest households have |*s*_0_(*p*)| ≪ 1.

The vector **s**′(*p*) contains the relative strengths of preference for various features of an individual dwelling and its environment—e.g., style of construction, number of rooms, location, fittings, access to amenities—and its match to actual features on offer in a dwelling contributes to that dwelling’s potential utility to the household, as discussed below. Ideally, all these components should be independent, but this is not essential; nor are the elements of **s** independent of those of **q**—e.g., a household of a particular religious characteristic might prefer a dwelling near their place of worship, or a large household might prefer a large dwelling—but they are expressed via two separate vectors because they affect the market dynamics differently, as we explain below. The element *s*_0_(*p*) governs price sensitivity and is assumed to be negative to impose a general preference for lower prices. This element is near −1 when the disposable income of *p* is low, thereby restricting other components of the unit vector **s**(*p*) to small values that leave little scope for other preferences to be exercised, as seen in Fig 1. We normalize so that the highest-disposable income household has *s*_0_(*p*) close to zero. These issues and that of the income distribution are further addressed in Sec. 4.

## 3 Utility function

We suppose that there are *M* dwellings, with *M* ≥ *N* to technically exclude homelessness, although dwellings may include temporary structures such as tents not normally occupied to ensure that every household has a designated dwelling, even if some would not qualify as dwellings for the purposes of defining homelessness under relevant social policies. (National Bureaus of Statistics often adjust dwelling counts for large regions and nationally, so that the total number of dwellings matches the total number of households in a census period, so *M* = *N* by definition.) Usually, *M* is approximately proportional to *N* [27], but construction implies that supply in growing areas of a city somewhat exceeds the number of households, so *M* > *N*.

The utility of a dwelling determines its price, subject to household income. When a transaction occurs, we equate the value of *U* (which includes a price penalty, as discussed below) with the price *P*, which is expressed in the form of the rent or interest payable, or the income foregone in a purchase. This means that all housing costs can be treated on the same basis as the income required to pay for them and measured in dollars per year or similar income unit.

**Fig 2:**
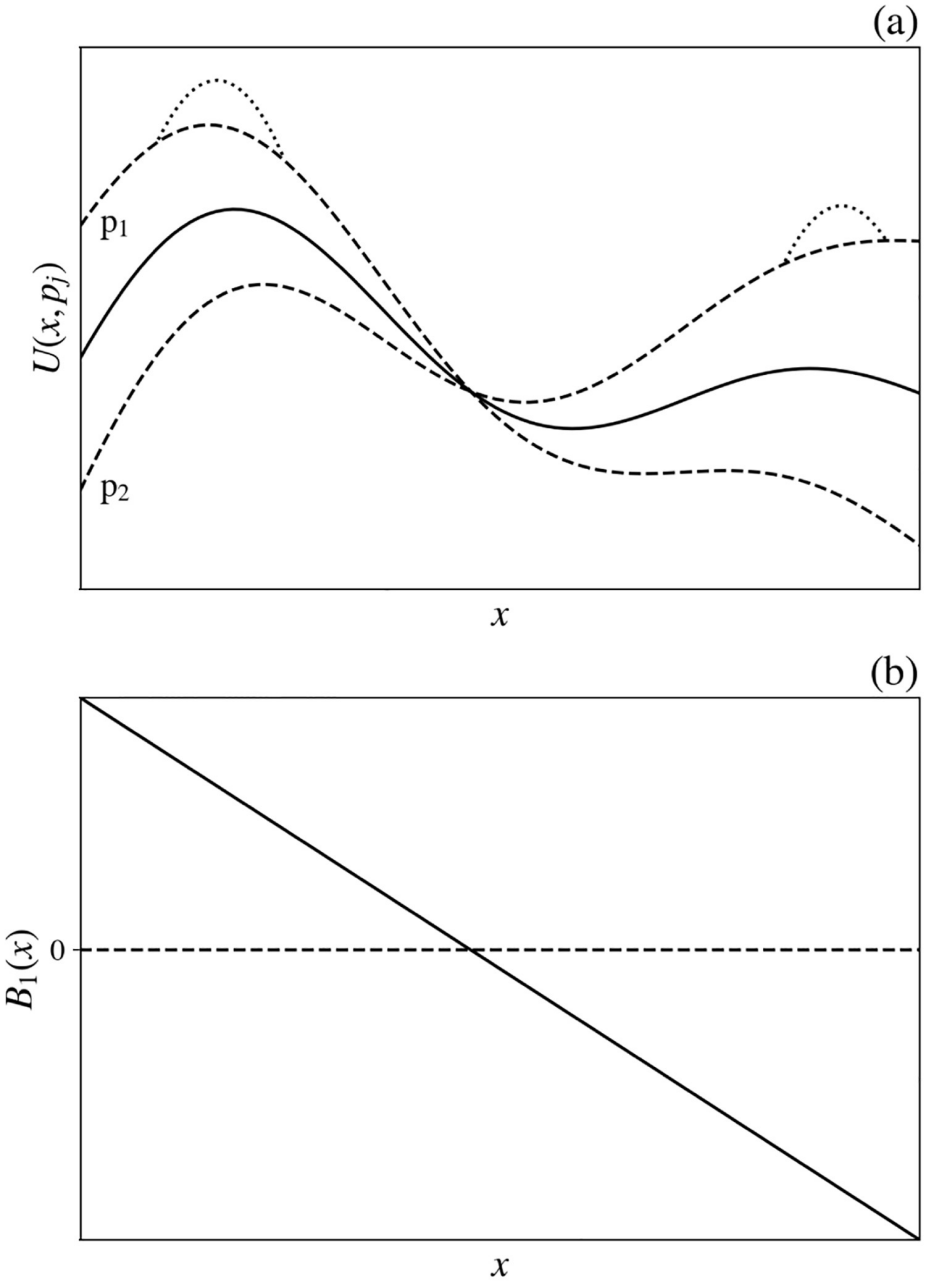
Schematic utility function *U* vs. dwelling position *x*, shown as one-dimensional for simplicity. (a) *U*_0_(*x*) is shown solid along with *U*(*x*, *p*_1_) and *U*(*x*, *p*_2_) (dashed) from Eq (4) for two households *p*_1_ and *p*_2_ with *s*_1_(*p*_1_) = 1 and *s*_1_(*p*_2_) = −1, to indicate like or dislike of characteristic *B*_1_, respectively. All other elements of the preference vectors are equal to zero. Dotted curves indicate the change in *U*(*x*, *p*_*j*_) if people of similar preference cluster near their utility maximum. (b) Spatial variation *B*_1_(*x*) vs. *x*.

A dwelling is distinguishable by its location **r**. The utility of a dwelling at **r** to a household *p* can be assumed to include a general utility *U*_0_(**r**) that is common to all potential residents [e.g., *U*_0_(**r**) is low next door to an open sewer or noisy factory], as illustrated in Fig 2(a). This term can also be used to incorporate restrictions or benefits imposed by geography or law; e.g., a prohibitively large negative *U*_0_(**r**) can be attached to flood plains, swamps, public parks, and other locations where dwellings are impossible or banned. Conversely, policies or market forces could favor particular locations by providing infrastructure that raises *U*_0_.

There is also a dwelling-preference contribution to utility that depends on the individual preference vector **s**(*p*), with

$$\begin{array}{c} U\left(r,p\right)=U_{0}\left(r\right)+s\left(p\right)\cdot B\left(r\right), \end{array}$$ (4)

where the dot denotes the usual dot (scalar) product and

$$\begin{array}{ccc} B(r) & = & [B_{0}\left(r\right),\ldots ,B_{n}\left(r\right)], \end{array}$$ (5)

$$\begin{array}{ccc} B^{\prime }\left(r\right) & = & [B_{1}\left(r\right),\ldots ,B_{n}\left(r\right)], \end{array}$$ (6)

are vectors of characteristics of the dwelling. Each component other than *B*_0_ can be positive or negative; for simplicity, these characteristics should be mutually independent, but this is not a requirement. To the extent that **s**(*p*) and **B**(**r**) align, this adds to utility, as shown in Fig 2(a).

**Fig 3:**
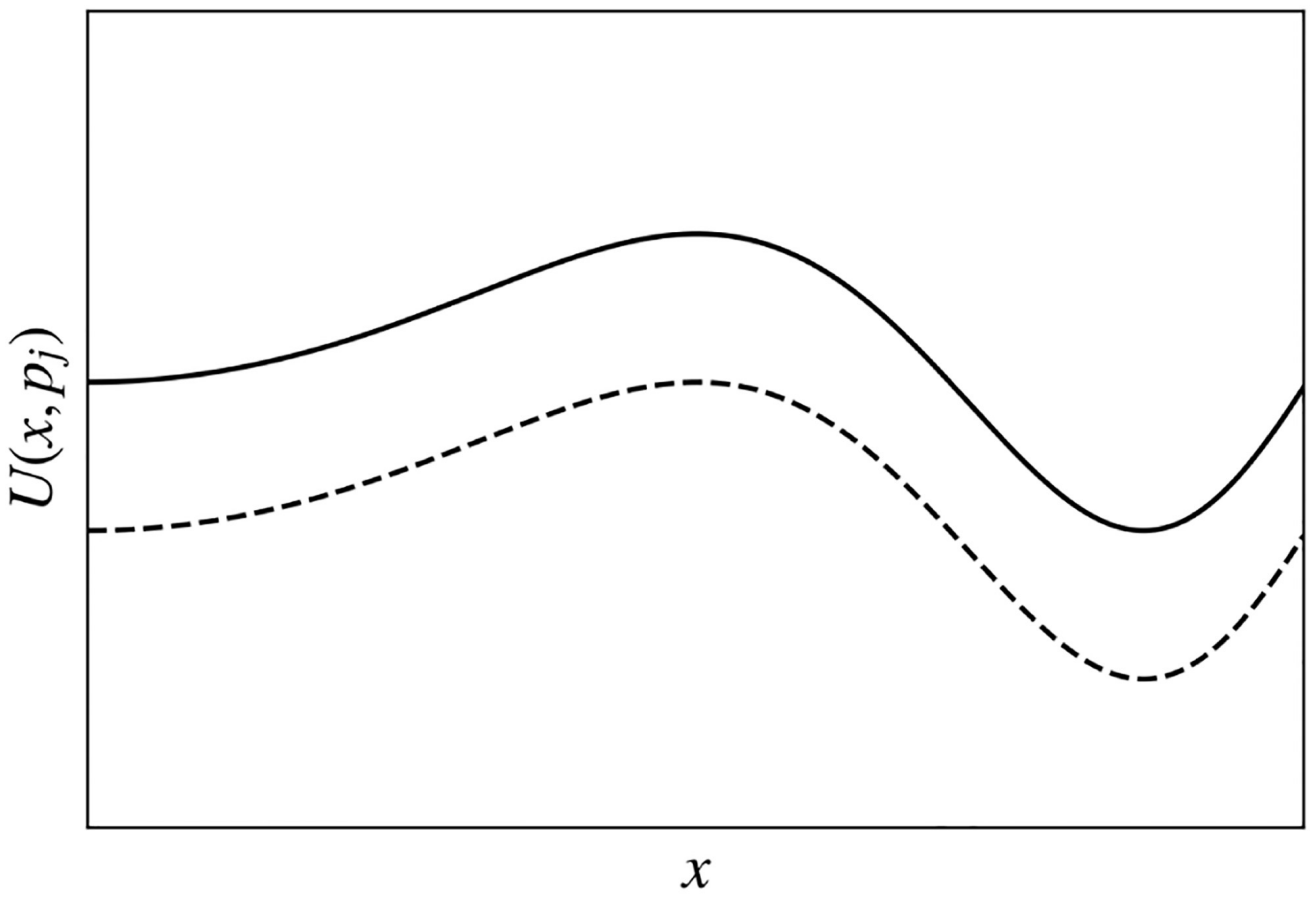
Schematic of the utility function *U*(*x*) vs. a one-dimensional position coordinate *x* for two different values of *s*_0_ with residual vectors s′ assumed parallel, so all preferences aside from price are in the same proportions. The upper curve is for a high-income household (*s*_0_ ≈ 0 and the lower one is for a low-income household *s*_0_ ≈ −1).

The component *B*_0_(**r**) (positive, with rare exceptions) is the cost of the dwelling at **r**, whose product with *s*_0_(*p*)<0 decreases utility (here we ignore cases where high prices are intrinsically favored by snobs), but less so for wealthy households whose price sensitivity *s*_0_ is relatively small (see Sec. 4). Note that: (i) Because housing cost is measured in income units, so are *U* and *B*_0_, whence *s*_0_ must be dimensionless; and (ii) this implies that all elements of **B** have the dimensions of money per unit time and all elements of **s** are dimensionless. Overall, Eq (4) describes the utility of a dwelling at **r** with characteristics **B**(**r**) to a household *p* with preferences **s**(*p*). Fig 3 shows how utility is lower for a household with low income (*s*_0_ ≈ −1) than for one with higher income (*s*_0_ closer to zero), when their preference vectors **s**′ are parallel.

Another contribution to utility is the preference of households for other residents with particular household characteristics, which we term the interaction utility. This modifies Eq (4) to

$$\begin{array}{c} U\left(r,p\right)=U_{0}\left(r\right)+s\left(p\right)\cdot B\left(r\right)+q\left(p\right)\cdot \sum_{p^{\prime }\neq p}q\left(p^{\prime }\right)G(|r-r\left(p^{\prime }\right)\left|\right), \end{array}$$ (7)

where the sum is over all *p*′ other than *p* itself and **q** is dimensionless. This equation is similar in structure to the energy of an assembly of interacting spins in an external field **B**, except that there are two distinct sets of spin vectors, labeled **s** and **q**. The dot product **q**(*p*) · **q**(*p*′) in the interaction utility quantifies the similarity in household characteristics between *p* and *p*′ and the the function *G* describes the strength of the preference (in units of money per unit time) for other households with similar characteristics and the range over which the interaction extends. Normally, *G*(0) will tend to be positive because people usually tend to like to live near similar people, and *G* will be a smoothly decreasing curve with a characteristic spatial width *h*, which can be viewed as the scale that sets the size of a neighborhood; in the simulations below we use

$$\begin{array}{c} G\left(x\right)=G\left(0\right)\text{exp}\left(-x^{2}/h^{2}\right), \end{array}$$ (8)

The dotted curves in Fig 2(a) illustrate the effect of contributions of this type via the final term in Eq (7) where it is assumed that existing residents with different signs of *s*_1_ are clustered toward the respective points of highest utility in the absence of this term.

A particular case of the interaction utility term in Eq (7) relates to the economies of scale and reduced transport costs that are achieved when households cluster together to form urban areas. We parameterize this effect through the term *q*_0_(*p*)*q*_0_(*p*′)*G*(|**r** − **r**)*p*′|), which could be approximated as not depending on *p* or *p*′ except through the proximity function *G*. This term will then be positive and will favor clustering and the formation of urban centers, but will be opposed by increased competition for housing and resulting higher prices, which reduce utility. If all dwellings are occupied, this term has no effect because there can be no change in clustering; however, it can drive choice when there are vacancies (see Sec. 7) and it also can help to drive the long-term construction of new dwellings by raising prices and profits, but we do not explore this aspect in the present work. Additionally, *U*(**r**, *p*) could include a shorter-range negative term of the same type in order to oppose excessive housing density as a type of zoning mechanism. This would result in an inverted Mexican-hat potential, with long-range attraction and hard-core repulsion between households.

More generally, the last term on the right of Eq (7) can be replaced by one of the form

$$\begin{array}{c} \sum_{p^{\prime }\neq p}\sum_{jk}q_{j}\left(p\right)G_{jk}(|r-r\left(p^{\prime }\right)\left|,p\right)q_{k}\left(p^{\prime }\right) \end{array}$$ (9)

where the function *G*_*jk*_ describes the contribution of the preference of *q*_*j*_(*p*) for *q*_*k*_(*p*′) at **r**(*p*) = **r**. A common case is where $G_{jk}(|r-r\left(p^{\prime }\right)\left|,p\right)=\delta_{jk}G_{j}(|r-r\left(p^{\prime }\right)\left|,p\right)$ and the functions *G*_*j*_ parameterize the relative importance to *p* of various characteristics of *p*′, not all of which will be significant and which may have different ranges. A case where *G*_*jk*_ might be nonzero for *j* ≠ *k* would be the preference of a low-income household to live in a high-income neighborhood; e.g., because of easier access to work or educational opportunities. On the other hand, a high-income household will typically not prefer a neighborhood dominated by low-income households. Significantly, *G*_*jk*_ has the dimensions of money per unit time, so it represents the value of particular types of neighbors in determining utility for household *p*. It may also help to estimate the level of funding that would be necessary to overcome pernicious segregation.

## 4 Price sensitivity and income distribution

There is a general preference to minimize housing cost, all else being equal. The income *m*(*p*) per unit time available for housing of household *p* is

$$\begin{array}{c} m\left(p\right)=m_{0}{\left[1-s_{0}^{2}\left(p\right)\right]}^{1/2}, \end{array}$$ (10)

where *m*_0_ can be chosen to be the maximal *m* in the population and −1 < *s*_0_ < 0. Here *m* includes the income equivalent of any relevant housing rental or purchase assistance and income support payments, which prevent the distribution from peaking sharply at or near *m* = 0. If there is a known income distribution *F*(*m*) the distribution of price sensitivity (i.e., preference) *s*_0_ is

$$\begin{array}{ccc} F(s_{0}) & = & F\left(m\right)|dm/ds_{0}|, \end{array}$$ (11)

$$\begin{array}{ccc} & = & F\left(m\right)\frac{m_{0}\left|s_{0}\right|}{\sqrt{1-s_{0}^{2}}}, \end{array}$$ (12)

where we omit the argument *p* for a population and *m* is to be written as a function of *s*_0_ in Eq (11) via Eq (10).

**Fig 4:**
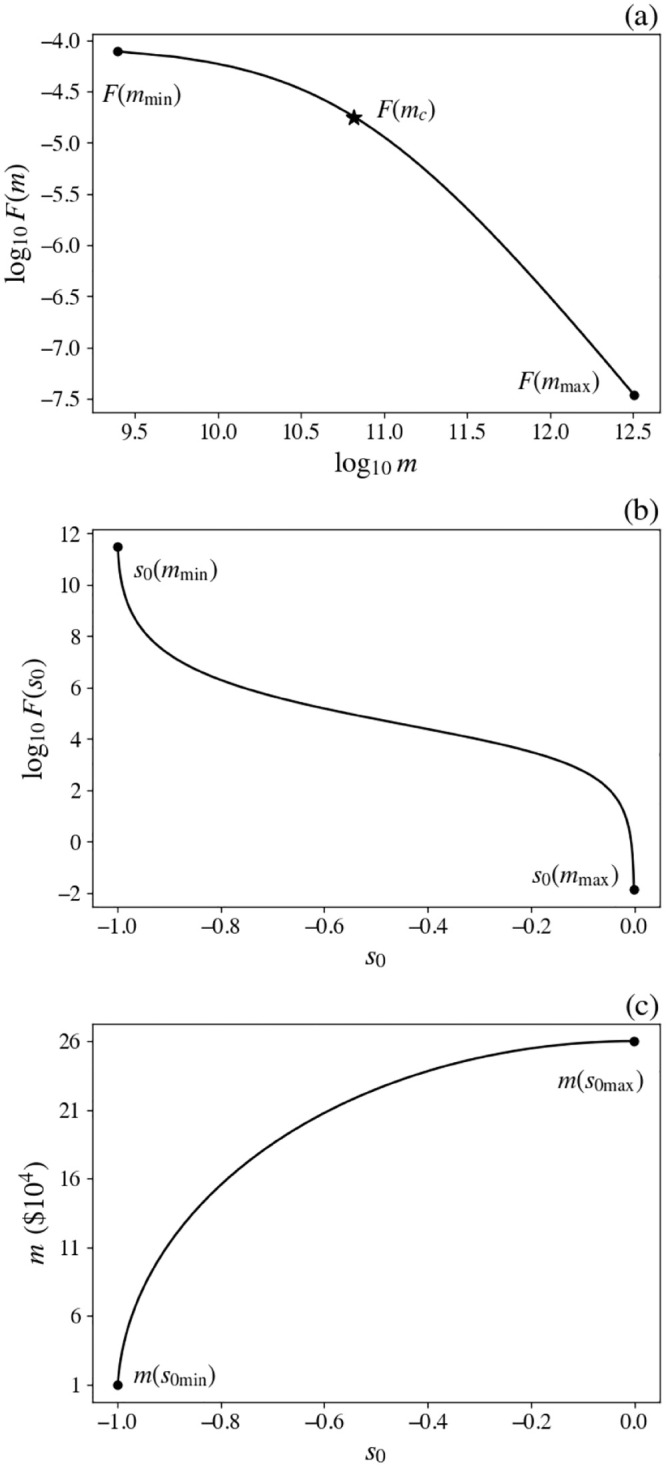
Schematic income distribution. (a) *F*(*m*) vs. *m* from Eq (11), income distribution *F*(*m*) (solid) showing the knee at *m*_*c*_ (star). (b) Same distribution vs. *s*_0_ from Eq (12). (c) Distribution of income *m* vs. *s*_0_.

A useful illustrative form of *F*(*m*) is

$$\begin{array}{c} F\left(m\right)=b{\left(1+m^{2}/m_{c}^{2}\right)}^{-a/2}, \end{array}$$ (13)

where *b* is a normalization constant chosen such that the integral of *F*(*m*) from *m* = 0 to *m* = *m*_0_ equals the total number of households, *m*_*c*_ is the point beyond which the distribution falls off rapidly, and *a* is is the exponent of a power-law tail at high *m*, with *a* = 2 in the present work. Note that the values of *a*, *m*_0_, and *m*_*c*_ imply *b*, as noted above, and must also yield the observed mean household income in the population, so they are not all independent. In this case

$$\begin{array}{c} F\left(s_{0}\right)=\frac{bm_{0}\left|s_{0}\right|}{\sqrt{1-s_{0}^{2}}{\left[1+\left(1-s_{0}^{2}\right)m_{0}^{2}/m_{c}^{2}\right]}^{a/2}}, \end{array}$$ (14)

for −1 < *s*_0_ < 0. Fig 4 shows an example of the corresponding distributions of *m* and *s*_0_, where we have normalized *F*(*m*) by adjusting *b* so that

$$\begin{array}{c} \int_{m_{\text{min}}}^{m_{\text{max}}}F\left(m\right)dm=N, \end{array}$$ (15)

where *m*_max_ and *m*_min_ are the maximum and minimum income levels in the population.

Once *s*_0_(*p*) has been chosen from a distribution such as the one in Eq (14), and the relative sizes of the components of **s**′(*p*) have been set, the latter must be normalized so that $|s^{\prime }\left(p\right)|=\sqrt{1-s_{0}^{2}\left(p\right)}$.

## 5 Determination of price from utility

**Fig 5:**
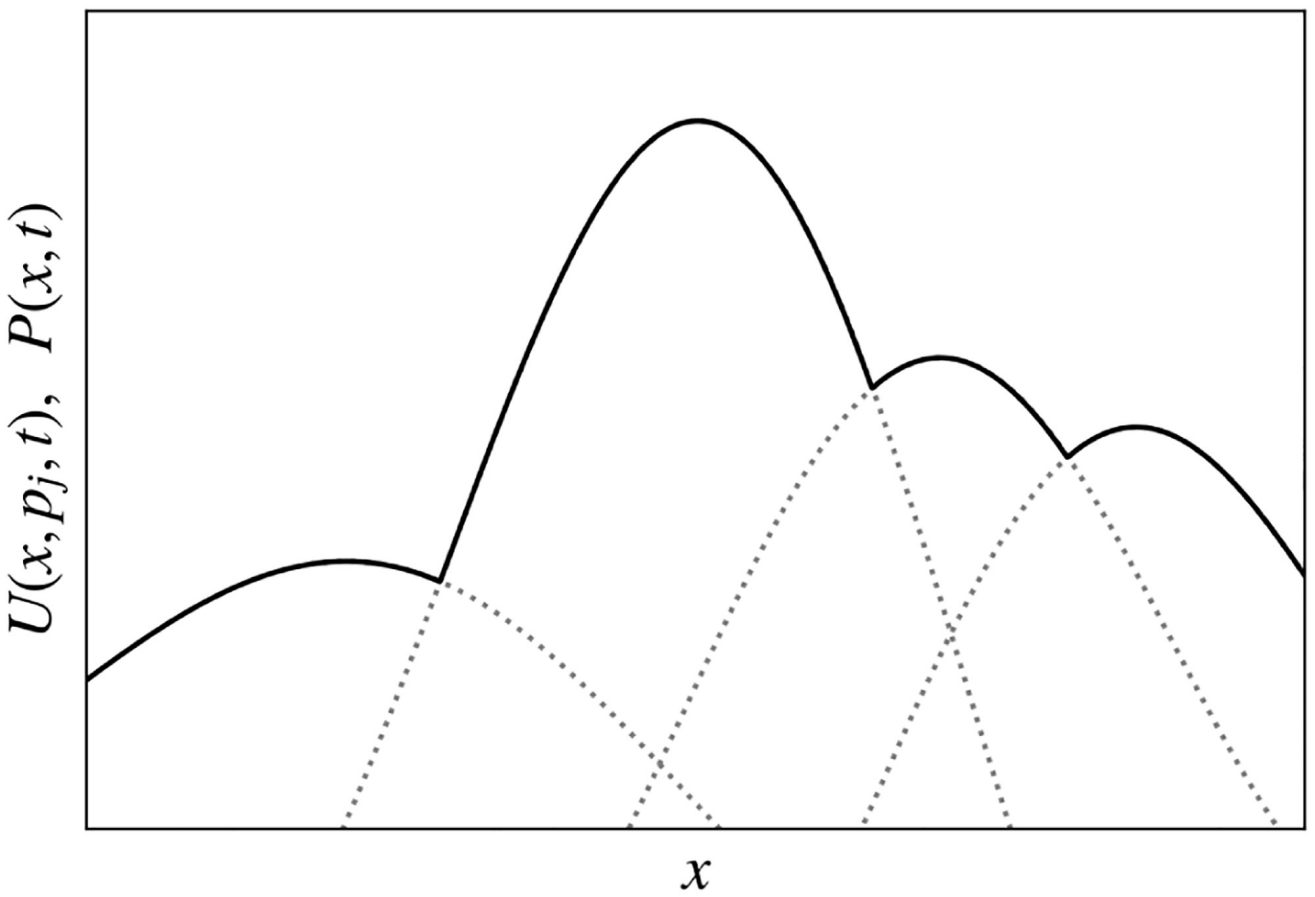
Schematic of how the utility functions of many households (dotted parabolas) determine the price function *P*(*x*, *t*) (solid curve), which is the upper bound of *U*(*x*, *p*_*j*_, *t*) over all *p* at time *t*.

As noted above, *U*(**r**, *p*, *t*) is the utility of a dwelling at **r** to a household *p* at time *t*, and hence what it would be worth to them per unit time if they could afford it. The income stream required to obtain (by purchase or rental) the dwelling at **r** is thus

$$\begin{array}{c} P\left(r,t\right)=\underset{r}{\text{max}}\;U^{\prime }\left(r,p,t\right), \end{array}$$ (16)

where *U*′ = *U*−*s*_0_*B*_0_ and the maximum is taken over all dwellings **r** that are on the market (see Sec. 6 for how this subgroup is determined), as shown in Fig 5. A negative price is possible, meaning either that dwellings are prohibited in a certain region, or that a dwelling is so undesirable that households would have to be paid to live there.

Transactions (housing sales or leases) are executed according to the following steps: (i) If a particular household *p* is the highest bidder at multiple values of **r** (e.g., if they are extremely rich) then any dwelling they actually acquire will be at a value of **r** (if any) at which their utility is both maximal and higher than that of their current dwelling; i.e., max_*r*_Δ*U*(**r**, *p*, *t*)>0 for such a household *p*. This avoids a situation where rich households acquire suboptimal dwellings just because they can easily afford them. If there are no dwellings available for which these conditions are met, then a household ceases to participate in the market. (ii) The transactions in (i) are completed first and *P*(**r**, *t*) is recalculated without those market participants and dwellings. (iii) Steps (i) and (ii) are repeated until all in-market households are housed or have failed to transact. (iv) The remaining dwellings can either remain on the market, be removed with some probability. It would also be possible to generalize the analysis to allow some households to remain in the market if they fail to transact, or even allow some to be unhoused, at the end of a given time step, but we leave these aspects for future work.

In future work one might incorporate an explicit model of property investment when excess disposable income is available. This would require a model of the wider economy, including investment returns and allocation between classes of investment, so we do not do so here. It would be easy, however, to distinguish owned and rented dwellings by an appropriate component of **B**.

## 6 Evolution of preferences, characteristics, supply, and demand

The actual market at any given moment does not involve all dwellings, nor all households; rather, only a subset of dwellings are on-market at any time, and a subset of households are looking to acquire dwellings. Here we consider the evolution of the numbers of active market participants. To do this we have to add time as an argument of all the functions introduced in the previous sections.

Here our aim is not to include all conceivable model elaborations and their combinatorial possibilities, but to provide a first approximation that can be employed to test core predictions of the model in the simulations described in Secs 7 and 8. We continue to mention other possible alternatives and generalizations, not all of which are investigated here, and further alternatives could doubtless be advanced in future. Ultimately, quantitative comparison with data will be needed to constrain model features and parameters.

### 6.1 Evolution of in-market population, and household preferences and characteristics

We incorporate population changes by generating (from initialized distributions) or deleting a random fraction *ϵ*Δ*t* of the **s** vectors at each timestep Δ*t*, on the assumption that completely new market entrants (e.g., who immigrate or attain adulthood) have roughly the same overall distribution of preferences as the existing population; alternatively, they could be assumed to have different average preferences based on age or immigration status. These enter the market, looking for housing but do not have dwellings to sell so they are assigned an in-market flag *μ*(*p*, *t*) = 1 (this flag is zero whenever *p* is off-market) but have no initial **r**(*p*, *t*).

Similarly a random fraction *ρ*Δ*t* of households is removed at each timestep, leaving their dwellings vacant. This fraction corresponds to death and emigration. These dwellings enter the supply with their current **r**(*p*, *t*) and *B*(**r**(*p*), *t*), with an on-market flag set to *ν*(**r**(*p*), *t*) = 1. Again, this process could be refined by allowing for the ages of the persons at various locations and adjusting the death and emigration rates accordingly. In the simulations below, we assume that households and dwellings don’t both enter and leave the market at the same time step, except through transactions.

A given household’s preferences are not static; for example, if *p*’s wealth changes, *s*_0_(*p*, *t*) varies accordingly. However, in the present work we treat the income distribution as static and *s*_0_ for new entrants to the market is chosen at random from this distribution. Likewise, deletion of market participants is random with respect to this quantity. Future refinements could include the effects of wealth generation, correlations of wealth with age, and other factors.

If *s*_0_(*p*, *t*) changes, the magnitude of **s**′(*p*, *t*) must also change to maintain |**s**(*p*, *t*)| = 1. Even at constant *s*_0_(*p*, *t*) it is possible for **s**′(*p*, *t*) to rotate within its *n*-dimensional subspace as relative preferences change due to factors such as ageing, technological advances, and social influences. The latter effect could be approximated after one timestep Δ*t* by adding an increment

$$\begin{array}{c} \Delta s^{\prime }\left(p,t\right)=-\alpha [s\left(p,t\right)-\langle s\left(p^{\prime },t\right)\rangle ]\Delta t, \end{array}$$ (17)

where the angle brackets indicate an average over a set of households *p*′ that could range from neighbors to the whole society; this term represents convergence toward common views at a rate *α*, which could be different for different components of **s**, more generally. After each timestep, **s**′(*p*, *t*) must be renormalized to ${\left[1-s_{0}^{2}\left(p,t\right)\right]}^{1/2}$ so that **s** remains a unit vector. Where the average in Eq (17) is over all of society, it would favor social uniformity, whereas if it is local it may contribute to fragmentation into subgroups [20–23].

Many other possibilities exist for contributions to preference evolution, including evolution toward greater preference for the attributes of one’s existing dwelling or of the average dwelling in its neighborhood.

In the present work we do not consider evolution of the household characteristic vector **q**(*p*, *t*) but it will change with time in general; at an absolute minimum the age of household members will change and this will drive changes in the preference vector **s**(*p*, *t*) due to changes in income and preference for different housing features at different ages. Likewise, household members and income will very often change with time.

### 6.2 Evolution of supply and characteristics of on-market dwellings

Section 6.1 discussed how dwellings are added to the supply when residents die or emigrate. A further source of supply here is that a fixed fraction *σ*Δ*t* of households put their dwellings on the market each time step if there is a location with higher utility than their current one; i.e., if

$$\begin{array}{c} \underset{r}{\text{max}}\;U\left(r,p,t\right)>U\left(r\left(p\right),p,t\right). \end{array}$$ (18)

One could make *σ* a function of the utility difference, for example, but we treat it as constant here. The dwellings that come on market through this process have the characteristics **B**(**r**, (*p*), *t*) as for deaths and emigrations and acquire the on-market flag *ν*(**r**, (*p*), *t*) = 1. For the present we do not consider major renovations and/or sudden changes in characteristics made prior to sale, but these could be incorporated in future. A change in supply also occurs when a dwelling is built from scratch, which we assume to occur by increasing the number of dwellings *M* by a fraction *β*Δ*t* of the *N*(*t*) occupied dwellings per time step. We use *N*(*t*) not *M*(*t*), the total number of dwellings, because *M*(*t*)>*N*(*t*) to include a buffer of unoccupied dwellings, as explained in Sec. 3 (these can include temporary shelters and transient structures) to ensure that everyone has a designated dwelling. Generally, we expect *β* should exceed the entry rate *ϵ* to allow for both renewal and expansion of existing building stock.

What is actually constructed is presumed to be above some threshold of unmet demand for utility. Because we do not model investment here, we approximate this by duplicating a fraction *τ* of the dwellings that have recently changed occupancy and assign them similar locations **r** + Δ**r** and similar characteristics

$$\begin{array}{c} B(r+\Delta r)=B(r,t)+\Delta B, \end{array}$$ (19)

where Δ**r** is a random vector with a characteristic size similar to the range *h* of *G* in Eq (7), so construction is in the same neighborhood, and |Δ**B**| is a random vector with magnitude equal to a fixed fraction of that of |**B**|. Alternatively, the increment Δ**B** could be taken to lie in the direction of ∇_**B**_*P*(**r**), the gradient of the price in the direction of **B**, to try to increase utility by better matching preferences expressed in the most recent transactions. The on-market flag of the newly constructed, unoccupied building is set to 1.

Other changes in supply occur when a building is demolished due to decrepitude or for potential profit, which we assume to occur for a random fraction λΔ*t* per timestep. In the case of demolition, the resident household *p* enters the market with its current preferences **s**(*p*, *t*) and *μ*(*p*, *t*) = 1 and the dwelling is replaced by a new one at the same location, on-market, with characteristics given by Eq (19). We expect λ will exceed the exit rate *ρ* to allow for renewal of dwelling stock. In the simulations below, we start with a substantial excess of possible dwelling locations to study the formation of urban areas from non-urban initial conditions without constraining where agglomerations will form, but set the demolition rate to a small positive value for dwellings that have not been occupied for a substantial amount of time to gradually reduce the excess. More details are given in Sec. 7.

A number of consequences will tend to follow from the above steps: (i) Concentrations will tend to rise because new dwellings are built near existing ones that have recently changed hands. This will tend to further raise local utility via Eq (7). (ii) Increases in utility due to changes in **B** will tend to push prices up, which will tend to oppose further increases in concentration. (iii) Population concentrations will tend to spread in space owing to the random increment Δ**r**, which will oppose the concentration effect in Eq (7), and because dwellings are cheaper on the outskirts.

Generalizations to improve the construction sector of our model would include better modeling building investment decisions by accounting for investors’ predictions of trends in household preferences, economic conditions, asset allocation between classes, projected profits, and resulting investment decisions. Factors like the business cycle, taxation policy, and interest rates affect the purchasing power of investors and buyers, and thus prices and transaction rates. However, incorporation of such effects would require a wider economic model that is beyond the scope of the present work.

Table 1 summarizes parameters discussed in the preceding sections and the values used in our simulations of a simplified version of the model. The upper part of the table shows dynamic quantities that mostly change in time, while the lower part shows parameters used in the various dynamic equations, along with their nominal values in our simulations, except where stated otherwise.

**Table 1:** Dynamic quantities and parameters shown above and below the line, respectively, with their symbols in the second column, nominal or initial values in the third, and units in the fourth, where $ denotes money. Dashes in the third column indicate values that depend on the details of the specific case chosen. Dashes in the final column indicate dimensionless quantities.

Quantity	Symbol	Value	Unit
Households	*N*(*t*)	1000	—
Household Preferences	**s**(*p*, *t*)	—	—
Household Characteristics	**q**(*p*, *t*)	—	—
Household In-Market Flag	*μ*(*p*, *t*)	—	—
Dwellings	*M*(*t*)	3000	—
Dwelling location	**r**	—	km
Dwelling Features	**B**(*r*, *t*)	—	$ y^−1^
Dwelling On-Market Flag	*ν*(**r**, *t*)	—	—
Utility	*U*(**r**, *p*, *t*)	—	$ y^−1^
Price	*P*(**r**, *p*, *t*)	—	$ y^−1^
System size	*R*	25	km
Neighborhood size	*h*	1	km
Neighborhood strength	*G*(0)	0	$ y^−1^
Fractional Entry Rate	*ϵ*	0	y^−1^
Fractional Exit Rate	*ρ*	0	y^−1^
Fractional Move Rate	*σ*	0.06	y^−1^
Fractional Build Rate	*β*	0	y^−1^
Fractional Demolition Rate	λ	0.02	y^−1^
Timestep	Δ*t*	0.25	y
Clearance Probability	*χ*	1	—
Income Exponent	*a*	2	—
Income Cutoff	*m*_*c*_	5 × 10^4^	$ y^−1^
Maximum Income	*m*_max_	2.5 × 10^5^	$ y^−1^
Preference Change Rate	*α*	0	y^−1^
Location Increment	|Δ*r*|	0.1	km
Feature Increment	|Δ**B**|/|**B**|	0	—

## 7 Numerical simulation

We now consider how to numerically simulate housing market dynamics using simple versions of the factors considered above. As noted above, many generalizations are possible—far too many to explore in a single publication. Our purpose is thus to explore a version of our model that is complex enough to display a variety of realistic effects, but which is simple enough to be tractable. This will enable us to demonstrate model’s utility via the initial applications in Sec. 8 and lay the foundation for future generalizations.

### 7.1 Model specification and initialization

The model in the previous sections requires us to consider the following: a list of households, *p*_1_, …, *p*_*N*_, each of which has a location **r**(*p*_*j*_, *t*) within a simulated spatial region; a preference vector **s**(*p*_*j*_, *t*) whose zeroth component is drawn from the distribution in Eq (14) with parameters *a*, *m*_*c*_, and *m*_max_; a characteristic vector **q**(*p*, *t*); and a flag *μ*_*j*_(*t*) = 0, 1 for each household which indicates whether or not it is in-market. New entrants to the market will not have an assigned location, so we set this to an arbitrary value that is well outside the simulated region. We also need a list of potential dwelling locations **r**_1_, …, **r**_*M*_, with *M* > *N*. The *k*th dwelling has a characteristic vector **B**_*k*_(*t*) and a flag *ν*_*k*_(*t*) = 0, 1 that indicates whether or not it is on-market.

Other parameters that must be specified are the rates *α*, *β*, *ϵ*, λ, *ρ*, and *σ* of the various processes discussed in Sec. 6, and the form of the function *G*, which we choose to be as in Eq (8). The default values listed in Table 1 are used in our simulations except where otherwise stated.

The initialization of the income distribution, and hence the distribution of price sensitivity *s*_0_, follows the process outlined in Sec. 4, using Eqs (14) and (15), for which the parameters from Table 1 give *m*_max_ = $250 000 y^−1^ and *m*_min_ = $11 000 y^−1^, respectively, with the mean of *m*(*p*) being $66 000 y^−1^. (Note that, although the symbol $ is used, we make no statement about which currency is used here, so an overall scaling is omitted.)

At the start of each simulation, all dwelling prices *B*_0_ are initialized to $0 y^−1^. Households are then randomly assigned to dwellings and those dwellings are taken off-market. We then calculate and update the price *B*_0_ of the newly occupied dwellings at *t* = 0 to be the price-free utility of the occupying household *U*′(**r**(*p*),0) = *U*(**r**,*p*,0) − *s*_0_(*p*,0)*B*_0_(**r**(*p*),0). This is the utility of the dwelling to the occupants if one neglects their ability to pay for it. If some low-income people are initially allocated to unaffordable housing, subsequent transactions enable them to move to dwellings they can better afford.

### 7.2 Time-stepping

**Fig 6:**
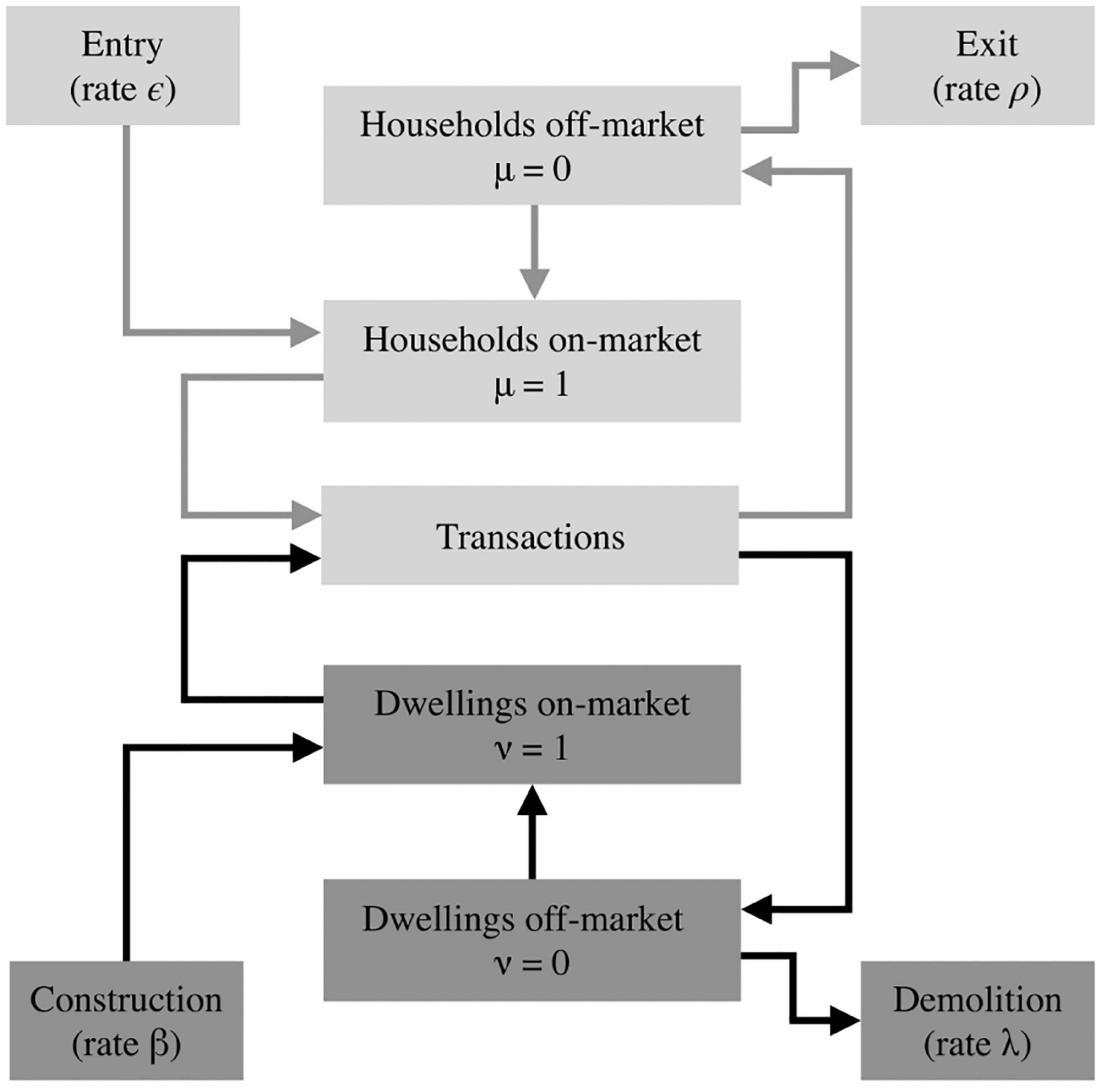
Schematic of how various changes in housing supply and population feed into transactions.

Putting together the material in the previous sections, we have the following procedure for advancing the dynamic quantities from time *t* to *t* + Δ*t*, as indicated schematically in Fig 6.

#### 7.2.1 Evolve housing/supply

(i) Demolish a fraction λΔ*t* of the current dwellings (randomly), removing them from the list and moving their occupants (if any) in-market with their current preferences and characteristics but no assigned location. Alternatively, one could demolish a fraction of dwellings as a function of age of the dwelling to allow for renewal of stock. In the present work, we set the initial number *M* of dwelling locations to be much greater than the number *N* of households to enable households to move significantly without having to implement a long initial period of dwelling construction for a gradually increasing population. However, such an excess of dwellings is not realistic and their presence increases the runtime of the numerical simulations. Hence, we gradually remove the excess by setting a nonzero probability that a dwelling will be selected for demolition, but only if it has been vacant for more than two years and only while *M* > 1.2*N*.

(ii) Build *β*Δ*tM*(*t*) new on-market dwellings by generating from an initialized distribution and assigning random locations and features according to Eq (6.2). Alternatively, one could have a profit-dependent *β* based on estimated utility and building cost, which would be spatially dependent in general.

#### 7.2.2 Evolve population and demand

(iii) Add a fraction *ϵ*Δ*t* of new entrants to the market by generating this fraction by duplicating members of the current distributions, but do not assign these households locations.

(iv) Delete a fraction *ρ*Δ*t* of households from the list and place their properties on market, if not already on-market.

(v) Place a fraction of all households in-market by setting *μ*(*p*, *t*) = 1 with a probability *σ*Δ*t*. For in-market households *p*_*j*_ calculate the utility *U*(**r**_*k*_, *p_j_*, *t*) from Eq (7) for all on-market dwellings *k* [i.e., with *ν*(*r*, *t*) = 1]. The probability of being placed in-market could also be defined as a function of utility.

#### 7.2.3 Evolution via transactions

Transactions remove households and dwellings from the market while increasing utility in the process; movements in **r** also result as a consequence. Transactions should occur on a shorter timescale than evolution of the building stock or population, so transaction steps could be carried out more often to shorten run-time, but in the present paper, transactions and updates of other quantities all occur quarterly.

The first task is to estimate the dwelling prices *B*_0est_ of the unoccupied and otherwise on-market dwellings. We don’t explicitly update the prices of all dwellings at each time step but rather estimate the price of the on-market dwellings prior to any transactions. This is essential to ensure that the dwelling price involved in a transaction approximates its true market value; otherwise market instabilities occur. In reality, such initial estimates are typically provided by sellers or their agents. Here, we generalize the *s*_0_*B*_0_ contribution in Eq (7) by noting that the price *B*_0_ has a individual-dwelling component *B*_0ind_ that is the *B*_0_ value of the dwelling determined from *U*′ without regard to the corresponding values for nearby dwellings, plus a component *B*_0ind_ that is a weighted average over the neighborhood to reflect factors such as land value—e.g., a decrepit shack could be located in desirable suburb. In the present work we use the same weight function as in Eq (8) and write

$$\begin{array}{c} B_{0\text{est}}\left(r,t\right)=xB_{0\text{ind}}\left(r,t\right)+\left(1-x\right)\left\langle B_{0\text{ind}}\left(r,t\right)\right\rangle , \end{array}$$ (20)

where 0 < *x* < 1 determines the relative individual and neighborhood contributions. This estimated dwelling price is then used to initialize the transaction process. In the examples below we set *x* = 0.5 on the basis that our numerical experiments show very similar results so long as *x* ≲ 0.99 is satisfied.

The next step is to evaluate the price function *P*(**r**, *t*) from Eq (16), where we do not include the *B*_0_ component, which is the price itself. The results tell us the most any household would like to bid for each on-market dwelling. We then use this value as an estimate of *B*_0_ in calculating the household’s total utility (now including the *s*_0_*B*_0_ term) to see how much they can actually afford to bid. Next, with probability *χ*, the in-market *p* with the highest income obtains the highest-utility dwelling they can afford for which their marginal utility Δ*U* is positive. If successful, they are removed from the market and their own dwelling goes on market at the next step. The price of their newly occupied dwelling is updated to equal the price free utility of the purchasing household. We set *χ* = 1 in the present work, but a probability *χ* < 1 could approximate some of the effects of market clearance delays, incomplete information, asynchronous transactions, and similar effects that prevent optimal matching of buyers and sellers.

If a transaction occurs as described in the previous paragraph, the price function is re-evaluated for remaining in-market households without the household *p* and the dwelling **r**(*p*) they acquired. Then the process is repeated in order of decreasing income *m*(*p*, *t*) [i.e., of increasing magnitude of price sensitivity |*s*_0_(*p*, *t*)|] until all possible trades have been completed. This may leave some unsatisfied bidders because higher-income bidders have purchased the only properties that would have increased the those lower-income households’ utility. These households stay in-market.

The above steps will tend to place the richest households in their most favored dwellings with a hierarchy of less-wealthy households in successfully less-satisfying dwellings. Alternatively, the order in which households transact could be made probabilistic vs. wealth, but we leave such extensions for future work.

#### 7.2.4 Evolution of preferences and housing characteristics

We do not simulate these aspects of the model in the present work. We note that on some timescale (not necessarily every step since these are slower processes) the preference vectors of households and characteristic vectors of properties can be evolved according to the rules in Sec. 6. This could also involve incorporation of inflation and/or decrementing the wealth of households who carry out a transaction to allow for taxes and transaction costs.

## 8 Tests and applications

In this section we present a selection of realistic applications to demonstrate the main features of our core model, showing that it can reproduce key real-world behaviors such as moncentric and polycentric city formation, income stratification, segregation, and balance between supply and demand, each emerging from the dynamics without being included a priori. Because there is combinatorial range of possibilities it is not possible to test every possible ramification, so our strategy is to examine proof-of-principle cases that can be generalized in future work. A variety of generalizations are also mentioned.

In the test cases below, all parameters are set to the values given in Table 1 unless otherwise stated. A general exception is that rates of household entry and exit and of dwelling construction are set to zero, as are the rates of evolution of preferences and characteristics (*ϵ* = *ρ* = *β* = *α* = 0). Since there is no construction, simulations are started with a substantial excess of dwellings, with *M* = 3*N*, and after two years unoccupied each such dwelling is demolished with a probability 0.02 y^−1^ until the number of dwellings is reduced to 1.2*N*; after this demolition ceases.

### 8.1 City formation

In this section we examine whether urban concentrations form spontaneously under the influence of economies of scale, as mediated by the *q*_0_ term in the household characteristic vector **q**, which indicates the presence of each household and favors clustering, plus the *s*_0_*B*_0_ term, which imposes a cost penalty on high clustering. We omit all household preferences for other characteristics of dwellings or neighboring households.

In the first test case, parameters are chosen so that the household characteristic vector **q**(*p*) is the scalar *q*_0_(*p*) = 1; the household preference vector **s**(*p*) is the scalar *s*_0_(*p*), drawn from the distribution in Eq (14); and the dwelling characteristic vector **B**(**r**) is the scalar *B*_0_(**r**). Dwelling locations are random within the overall simulation area and, as mentioned in Sec. 7.1, *B*_0_(**r**) = 0 initially for all dwellings. We also set the general utility *U*_0_(**r**) = 0 in this example so there are no preferred locations and the geography is that of a featureless plain.

**Fig 7:**
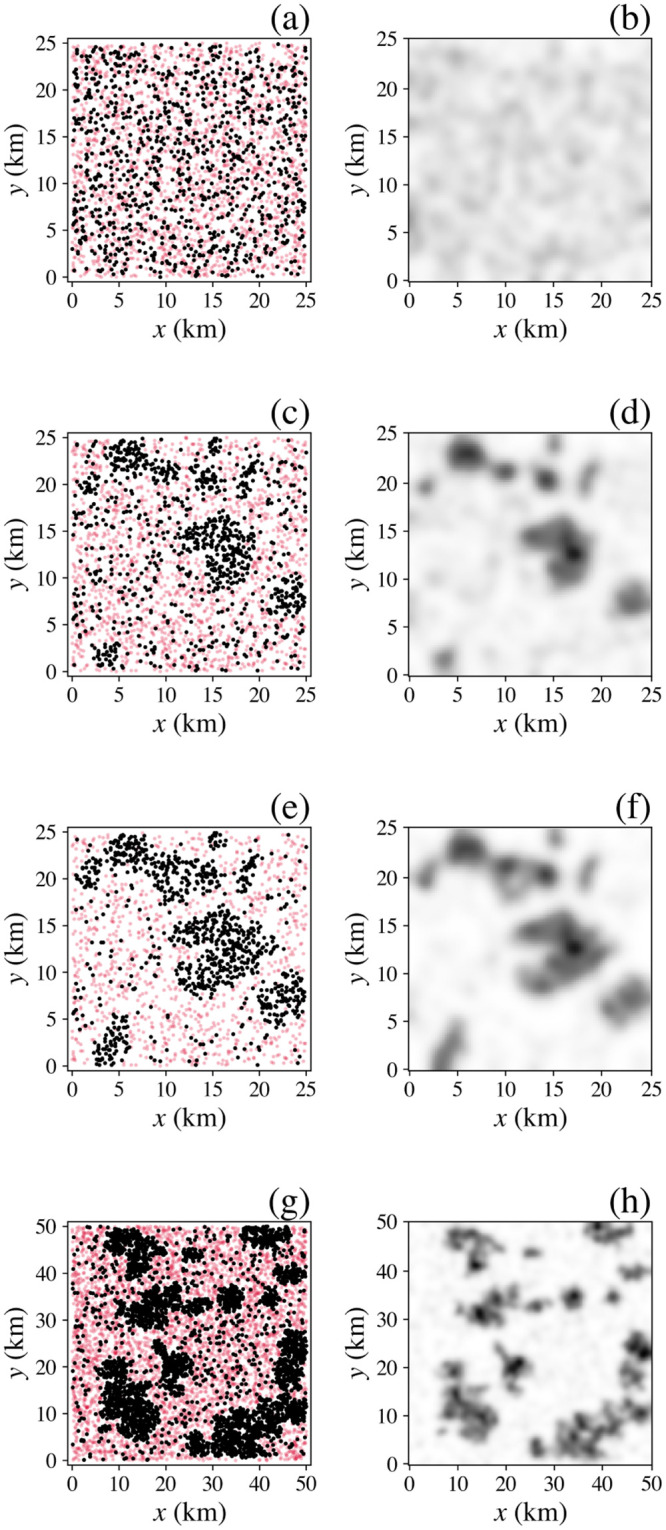
City formation and clustering effects. Spatial distribution (left) of households (black) and dwellings (red) and smoothed distribution (right, same grayscale in all frames) of households at various times: (**a**) and (**b**) *t* = 0 years, (**c**) and (**d**) 10 years, (**e**) and (**f**) 25 years. (**g**) and (**h**) Results for a larger system with *R* = 50 km and the same initial density of dwellings and households. Note that colors appear denser in (g) because four times as many points are plotted.

In Fig 7(a), 7(c), and 7(e) we see that over 25 years households (black dots) gradually form three or four main clusters (the number depends on how their boundaries are defined), which we can identify as settlements, each with internal structure, because the final term on the right of Eq (7) favors agglomeration; dwelling locations (shown in red) are fixed. Of course, the numbers of dwellings and households in real cities is much larger than here, but each can be taken to be representative of a larger number; this restriction can be relaxed at the cost of longer computational runtime.

Fig 7(b), 7(d), and 7(f) show the corresponding densities of households, smoothed by convolving with a Gaussian kernel of standard deviation 3 km. A movie of this evolution is found in the S1 Video. Fig 7(g) and 7(h) show the corresponding results for a system that is identical except for having twice the linear extent (*R* = 50 km) in each dimen-sion and four times the total number of households and dwellings to keep their initial areal densities the same. We see that clusters form with a variety of sizes. Each is irregular in shape, reminiscent of real urban areas, even though there are no geographical features such as rivers, coasts, or highways here to constrain their structure. Hence, the model produces qualitatively realistic polycentric structure. Conversely, we find that in a smaller system relative to the neighborhood size *h* a single cluster forms (not shown), corresponding to a monocentric set-tlement. Future extension of the model to incorporate transport costs might be expected to fur-ther favor polycentrism to avoid the need for excessively long trips to a single central business district; however, this will be opposed by historic entrenchment of some employment and governmental functions in the initial CBD. Likewise, recent pandemic-driven increases in home-based work may tend to flatten urban density.

### 8.2 Effect of *U*_0_

**Fig 8:**
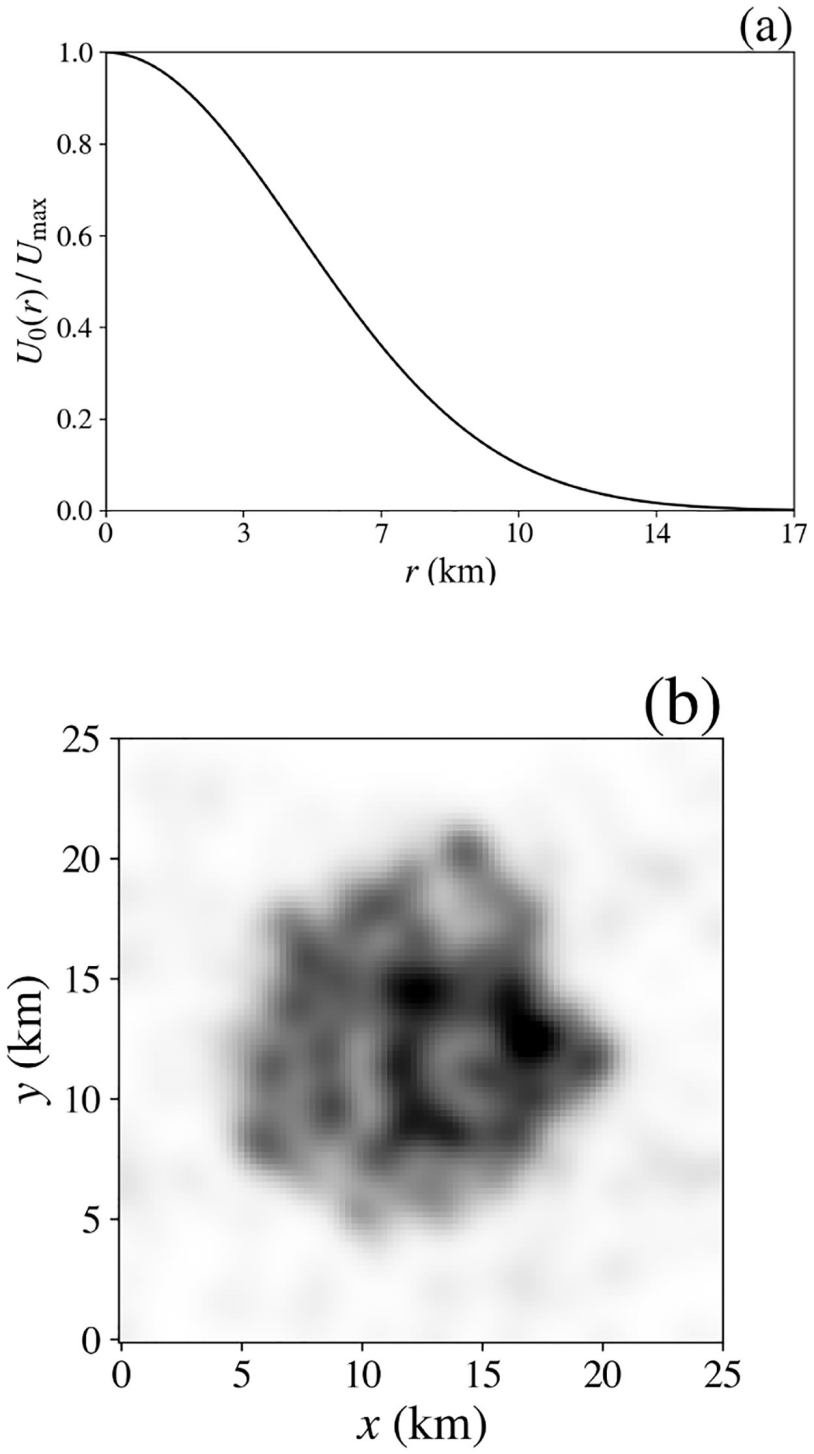
City formation around a central point of maximal *U*_0_. **(a)** *U*_0_(*r*)/*U*_max_ vs. distance *r* from the center from Eq (21). **(b)** Spatial distribution of households after 25 years.

Here we show that a naturally favored region—e.g., a water supply, employment center, or transport nexus—can lead to the formation of a surrounding settlement. In this case, the general utility *U*_0_ is set to have a peak of *U*_0max_ = $5 × 10^3^ y^−1^ at the center of the simulated area, declining to a negligible value at the edges, with

$$\begin{array}{c} U_{0}\left(r\right)=U_{0\text{max}}\text{exp}[-|r-r_{0}{|}^{2}/{\left(\Delta x\right)}^{2}], \end{array}$$ (21)

where **r**_0_ = (12.5, 12.5) km is the location of the maximum and Δ*x* = 7 km is its characteristic width, as seen in Fig 8(a). The vectors **q**, **s**, and **B** are the same scalars as in Sec. 8.1 and the simulation parameters are the same with random dwelling location and initially random household assignment. In Fig 8 (b), the spatial density of households at the end of the 25-year simulation shows the formation of a single cluster, centered at the point of maximal *U*_0_(**r**), thus demonstrating the ability of the model to produce a monocentric city.

**Fig 9:**
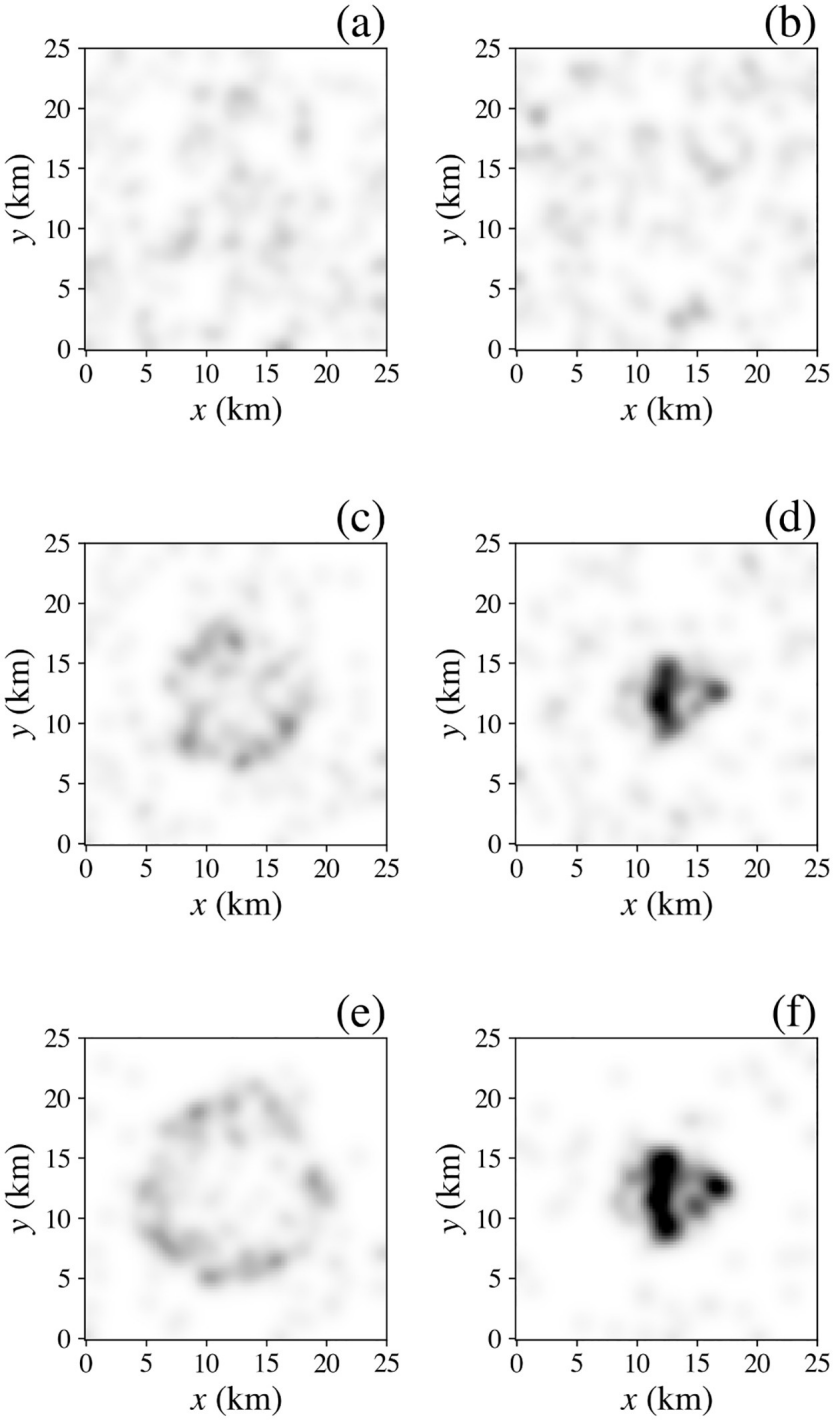
City formation around a maximum of *U*_0_ at (12.5, 12.5) km, shown at various times. The left and right columns show smoothed distributions of the lowest 20% and highest 20% of households by wealth, respectively, with the same grayscale in all frames. (**a**) and (**b**) *t* = 0. (**c**) and (**d**) *t* = 10 y, (**e**) and (**f**) *t* = 25 y.

A further feature of the dynamics is that households stratify according to income, with the richest concentrated closest to the maximum of *U*_0_ due to their lower price sensitivity. This effect is evident in Fig 9, where the left and right columns show snapshots of the spatial distributions of poor and rich households, respectively, from the simulation in Fig 8. As time progresses the rich accumulate at the center, where the higher density adds to utility, whereas the poor spread to form a ring on the outskirts of the city where housing is cheaper, as seen in real cities. Future extension of the model to include transport costs can be expected to change this picture somewhat: greater car ownership among the wealthy can facilitate those who prefer suburban lifestyles to commute between an outer ring and the center, whereas others may concentrate in a gentrified center, leaving the poor at intermediate distances. Alternatively, if there is insufficient profit to be made in redeveloping the inner city, urban decay can lead to the poor being concentrated centrally, often alongside commercial enterprises and employment hubs, with the wealthy living further out. An extension of the present work to incorporate a model of investment will be needed to explore these possibilities.

**Fig 10:**
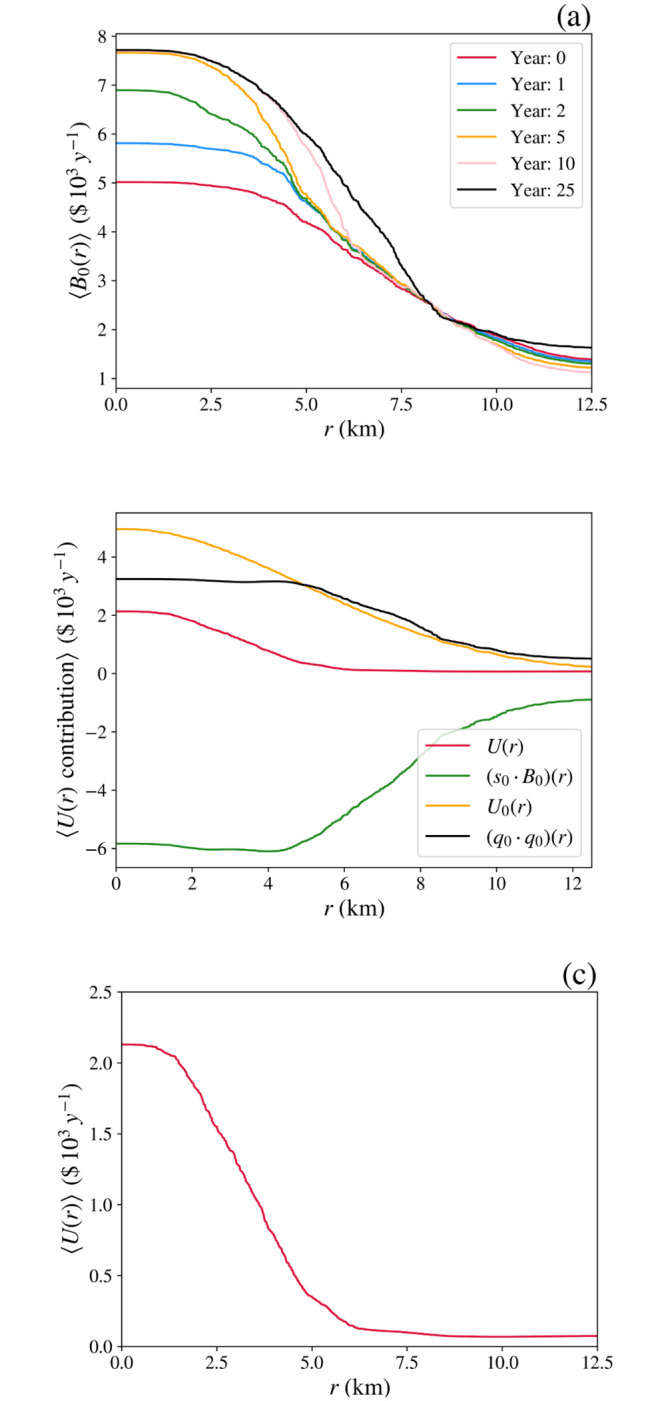
Evolution of contributions to utility vs. distance r from the peak of *U*_0_, each curve smoothed to remove short-scale fluctuations for clarity. (**a**) Mean dwelling price *B*_0_(*r*) at various times indicated by the legend. The plot at *t* = 0 years occurs just after dwelling assignment; prior to dwelling assignment, *B*_0_ is uniformly zero and is not shown explicitly. (**b**) Contributions to mean household utility *U*(*r*), as indicated by the legend. (**c**) Expanded view of mean household utility *U*(*r*) as a function of distance r from the maximum of *U*_0_ at (12.5, 12.5), as indicated by the legend.

During the simulation in Figs 8 and 9, the average dwelling price 〈*B*_0_(*r*)〉 (angle brackets denote an azimuthal average at constant *r*), seen in Fig 10(a) as a function of the distance *r* from the center, starts at large *r* with a nonzero value that arises via the *q*_0_(*p*)*q*_0_(*p*′) density-dependent interaction utility in Eq (7) evaluated at the initial mean household density. It then rises to central maximum that reflects the additional effect of *U*_0_. As time progresses, wealthy households move to the central region, which increases the interaction utility there and causes a slight decrease in the surrounding countryside where the population density falls. The profile becomes flat-topped once all the dwellings in the central region are occupied and no further increase in the interaction utility is possible. In the real world, such a situation would likely drive investment in construction in the central region to exploit the potential value of still higher densities, but we do not explore this aspect in the present work.

The mean total utility 〈*U*(*r*)〉 after 25 years, shown by the red curve in Fig 10(b), is the sum of several contributions. First, *U*_0_(*r*) has the form given by Eq (21), peaking at $5000 y^−1^. To this is added the density dependent term, which declines from $3242 y^−1^ at the center to $512 y^−1^ in the countryside. Largely offsetting these is the disutility caused by price 〈*s*_0_*B*_0_〉 which has a negative peak of −$6097, *decliningto* -92 in the countryside. The magnified plot of mean total utility in Fig 10(c) shows the resulting peak of only $2130 y^−1^ and a minimum of $69 y^−1^. Interestingly, this implies that the cost of housing negates the bulk of the intrinsic utility when there is strong competition, so a contrarian household with very different preferences can potentially obtain a bargain; however, in the long term, the supply of different types of housing would be expected to evolve to reduce this effect.

The relatively small total utility implies that provision of modest general utility at a new location might well lead to large effects on population distribution. Examples include the establishment of capital cities such as Canberra and Brasilia ex nihilo, which initiated substantial migration to their locations. Likewise, construction of a new mine or factory would have similar effects.

### 8.3 Effects of spatial localization of favored dwelling characteristics

We now investigate the case in which a particular favored dwelling characteristic is spatially localized; this could model preference for location near a lake, an employment or retail hub, or a school, for example. Hence we introduce a two-component household preference vector **s**(*p*) = [*s*_0_(*p*), *s*_1_(*p*)] and corresponding dwelling characteristic vector **B**(**r**) = [*B*_0_(**r**),*B*_1_(**r**)], while *q*(*p*) remains the scalar *q*_0_(*p*). To model a localized peak, we choose the form

$$\begin{array}{c} B_{1}\left(r\right)=B_{1\text{max}}\text{exp}[-|r-r_{1}{|}^{2}/{\left(\Delta x_{1}\right)}^{2}]. \end{array}$$ (22)

with Δ*x* = 3 km, with the household preferences *s*_1_(*p*) for this characteristic randomly sampled from a uniform distribution over the range [−1, 1].

**Fig 11:**
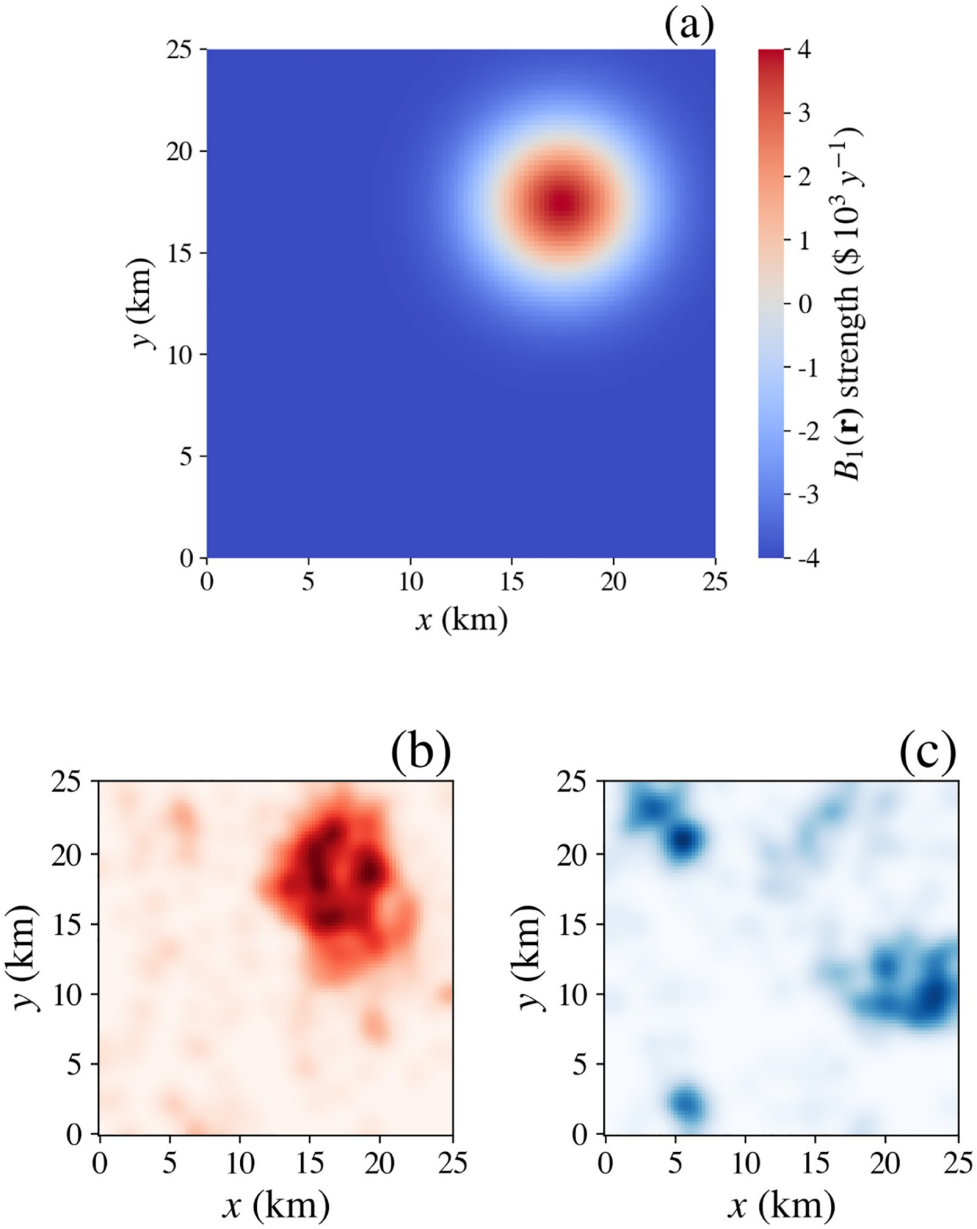
Effects of a spatially localized dwelling characteristic *B*_1_ and after 25 years. (a) *B*_1_(**r**) vs. **r** with a spatial maximum at (17.5, 17.5) km. (**b**) Distribution of households with *s*_1_ < 0 that seek positive *B*_1_. (**c**) Distribution of households with *s*_1_ > 0 that avoid positive *B*_1_.

The results of a 25-year simulation in which all other parameters are the same as in Table 1 are shown in Fig 11 for **r**_1_ = (17.5, 7.5) km and *B*_1max_ = $4000 y^−1^ to obtain a significant effect. Fig 11(a) shows the form of *B*_1_(**r**), while Fig 11b shows that households with a preference for *B*_1_ (i.e., those with *s*_1_ < 0) cluster around the peak of *B*_1_(**r**). Households that have *s*_1_ > 0 dislike the characteristic *B*_1_ and instead form clusters in regions where *B*_1_ is small, as seen in Fig 11(c).

Many variants of the case considered here can be envisioned, modeling preferences for features such as environment, facilities, and employment opportunities. The effects of competing preferences, especially ones that cannot be simultaneously satisfied, could also be examined.

### 8.4 Segregation

A key feature of many urban areas is segregation on the basis of household characteristics such as ethnicity, religion, or occupation (e.g., farmers vs. office workers), which can be viewed as components of the household characteristic vector **q**(*p*). In addition to *q*_0_ we thus assume that each household has a randomly assigned binary characteristic *q*_1_(*p*) = ±|*q*_1_| so similar neighboring households increase utility via Eq (7) and dissimilar ones reduce it. To focus on segregation, we revert to **s** and **B** being scalars with only zeroth elements retained to embody price sensitivity.

**Fig 12:**
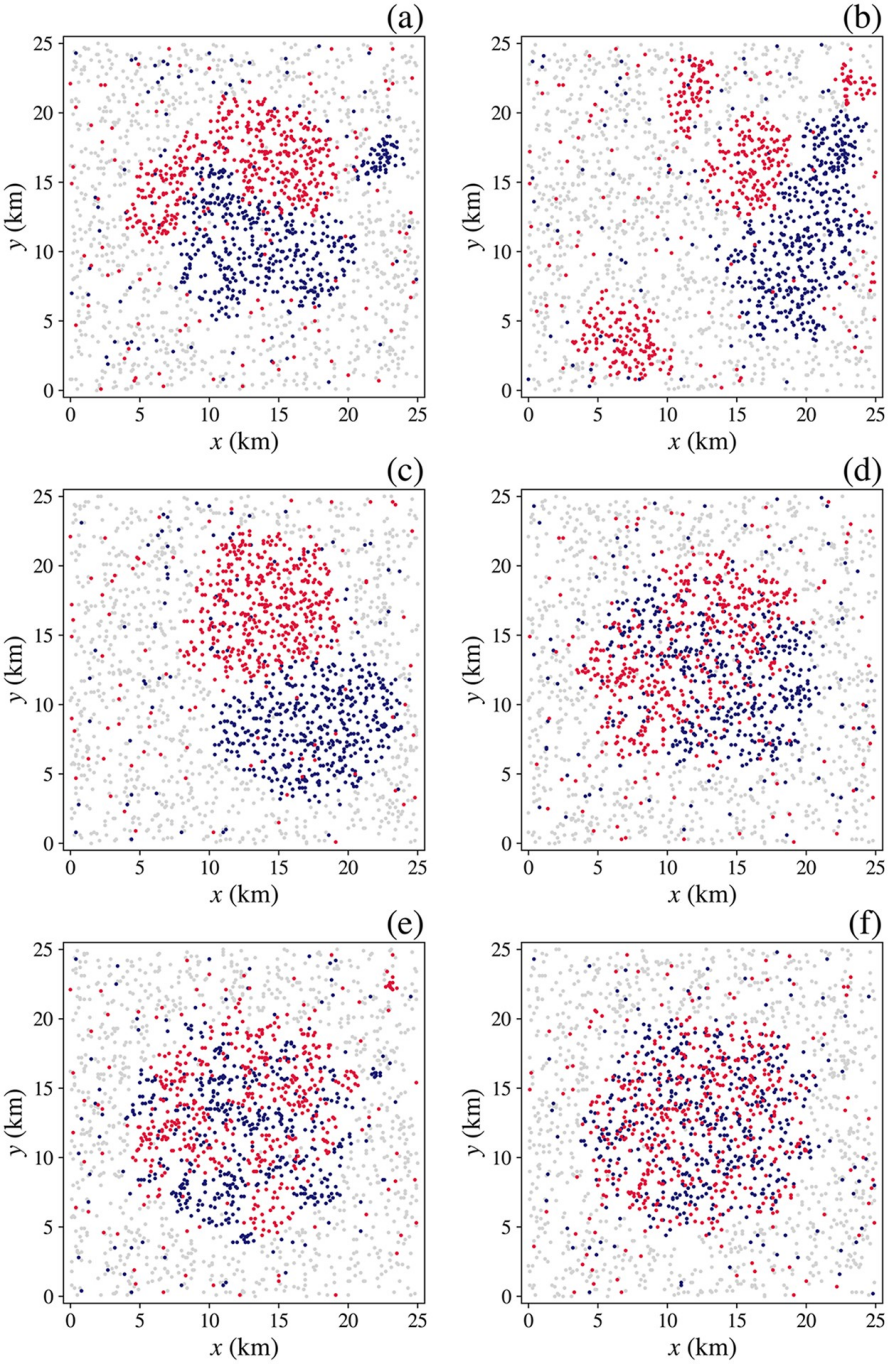
Segregation after 25 years of equal numbers of households with *q*_1_ = 1 (red) and *q*_1_ = -1 (blue). Dwellings have general utility *U*_0_ distributed as previously centered at (12.5, 12.5). Characteristic type *q*1 and neighborhood size *h* are set to the following combinations: (**a**) *h* = 1 km, *q*_1_ = ±1, (**b**) *h* = 1 km, *q*_1_ = ±2, (**c**) *h* = 2 km, *q*_1_ = ±1, (**d**) *h* = 1 km, *q*_1_ = ±0.5, (**e**) *h* = 0.5 km, *q*_1_ = ±1, (**f**) *h* = 1 km, *q*_1_ = ±0.25.

In the first case considered, we explore the effects of the size of *q*_1_ (i.e., the strength of preference for similar neighbors) and the range *h* of the kernel function in Eq (7), which characterizes how far away dissimilar neighbors need to be before they are effectively ignored. We specify a point of maximal *U*_0_ at coordinates (12.5, 12.5) km as in Sec. 8.2, with other parameters as in Table 1. Fig 12(a) depicts the resulting overall central clustering for *h* = 1 km and *q*_1_(*p*) = ±1, as in Fig 8, but now with azimuthal segregation by household type into two zones with a fairly sharp interface and little interpenetration. The population is more homogenous on the outskirts where distances between households are greater and there is less interaction. Case (b) is the same except that the preference strength has been increased to *q*_1_ = ±2. In this case, the mutual antipathy of the two groups has forced them both away from the peak of *U*_0_, thereby reducing both groups’ general utility—so intolerance harms everyone, including the intolerant. Fig 12(c) shows that a similar effect occurs for *h* = 2 km, with this longer-range mutual intolerance forcing the two communities to live further apart. Conversely, Fig 12(d)–12(f) show that a reduction in the strength and/or range of the mutual antipathy quickly leads to more integrated communities and higher general utility. Notably, the contribution of the *q*_1_(*p*)*q*_1_(*p*′) interaction term in Eq (7) to the utility is a measure of how much utility households will forgo to satisfy their intolerance; it also provides an estimate of how much countervailing utility would need to be provided to overcome this effect. Notably, if one group is wealthier than the other it will dominate the higher-utility areas, pushing the poorer group into less-favorable regions, as was the case in Fig 9.

**Fig 13:**
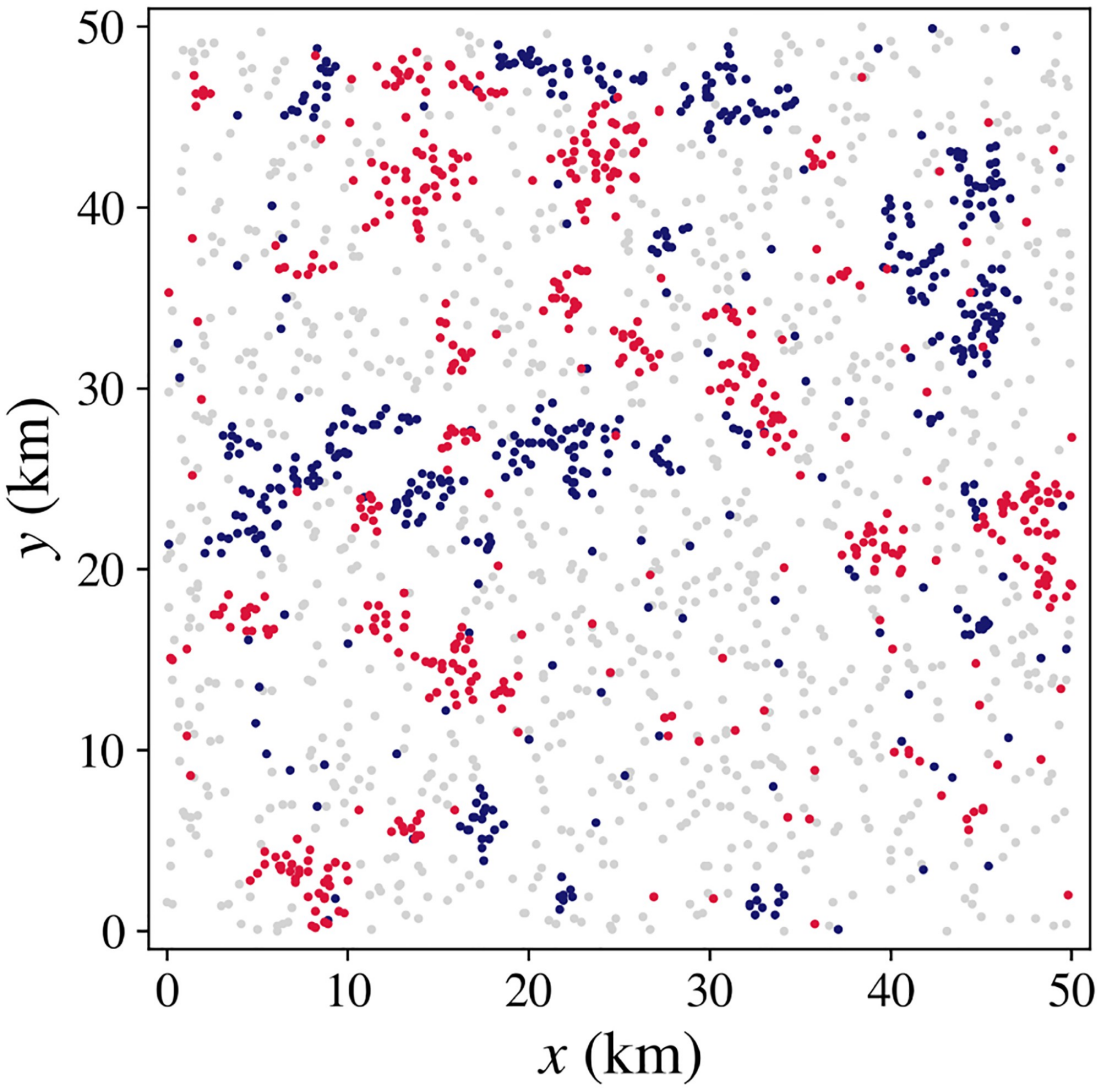
Segregation after 25 years of equal numbers of households with *q*_1_ = ±1 with neighborhood size *h* = 1 km, system size *R* = 50 km, and *U*_0_ = 0.

The example shown in Fig 13 we initialize a large system with *R* = 50 km with *U*_0_ = 0 and random initial locations, as in Fig 7 in Sec. 8.1, but with *h* = 1 km and *q*_1_ = ±1 as in Fig 12(a). Fig 13 shows a similar clustering to Fig 7(g), but the clusters are either overwhelmingly of one type, or are internally segregated into homogeneous neighborhoods if large enough. A movie of the evolution of this example can be found in the S2 Video.

It has been argued that segregation can arise purely from a liking for similarity, without a dislike of a dissimilar group [11]. The incorrectness of this argument can be seen by writing the matrix of interactions of *q*_1_(*p*) and *q*_1_(*p*′) in the form

$$\begin{array}{c} (\begin{array}{cc} 1 & -1\\ -1 & 1 \end{array}), \end{array}$$ (23)

for two groups that are mutually antipathetic. Here the axes represent groups, ordered from the top left, the on-diagonal positive entries represent preference for similar neighbors, and off-diagonal negative entries represent negative preference for dissimilar ones. The assertion that only positive preferences are involved in some cases, with neutrality toward dissimilar neighbors, can be dispelled by writing the corresponding preference matrix in the form

$$\begin{array}{c} (\begin{array}{cc} 1 & 0\\ 0 & 1 \end{array})=\frac{1}{2}(\begin{array}{cc} 1 & 1\\ 1 & 1 \end{array})+\frac{1}{2}(\begin{array}{cc} 1 & -1\\ -1 & 1 \end{array}). \end{array}$$ (24)

What this shows is that the asserted positive preference (at left) can be written as the sum of a preference for people in general (the first term on the right, which belongs in the *q*_0_ component of the interaction utility) and a mutual-hostility contribution of the form discussed above (the second term on the right); the coefficients of 1/2 are not significant because overall normalization of **q** has not been applied. Thus, preference toward one group implies *relative* hostility toward the other.

A possibility for future investigation is that there may be a threshold for segregation, such that inclusion of sufficiently strong shared characteristics in **q** may prevent segregation, unless one characteristic is a dominant consideration for certain groups, because it is impossible to have a separate neighborhood for each combination of characteristics. In this case, encouraging a variety of mutual interests and preferences (but not conformity to just one set) would tend to bind communities together, without suppressing differences. Over time, the degree of commonality would likely increase to produce the “melting pot” effect sometimes observed; such evolution of household characteristics **q** toward a community mean can be incorporated in the current model.

Other areas for investigation would be to include correlations between group status and wealth, unequal numbers of the two groups, more groups, and/or different levels of antipathy between groups. Continuously valued household characteristics could also be used to model the fact that not all members of a group view other groups in the same way.

### 8.5 Supply and demand

**Fig 14:**
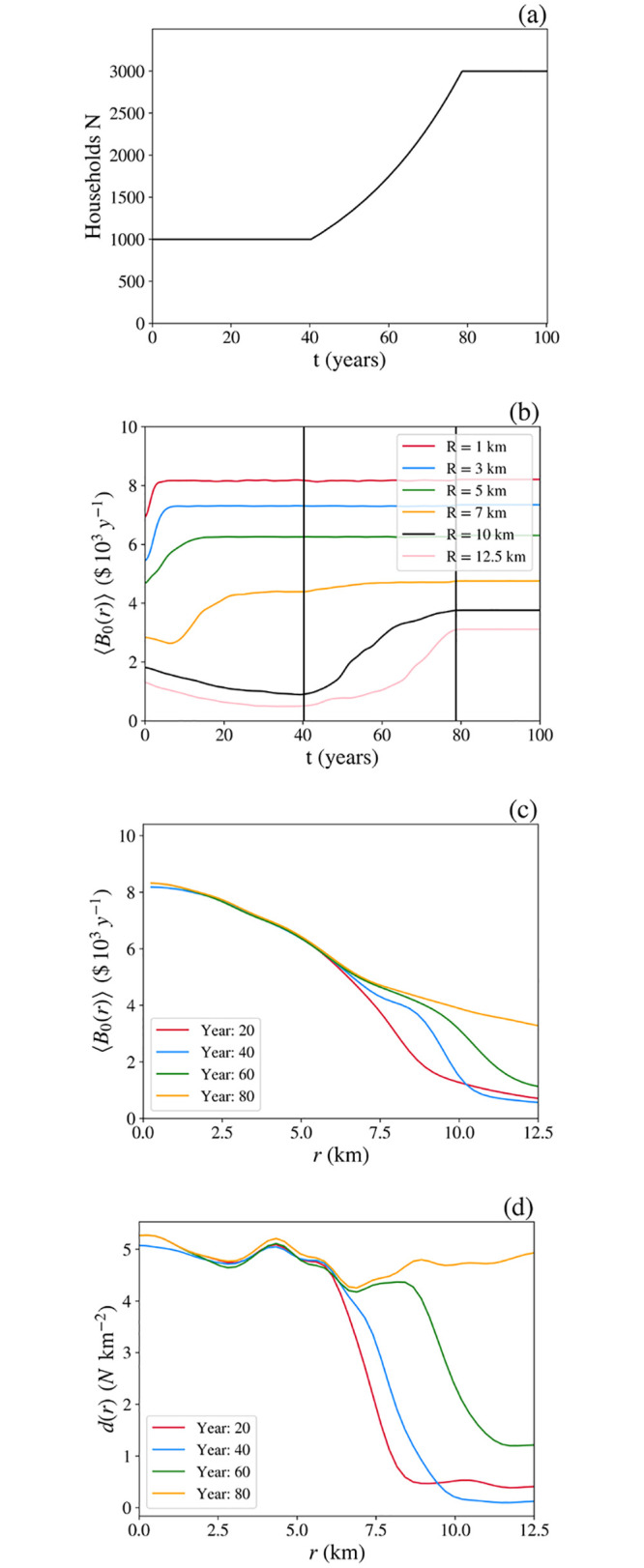
Supply-demand dynamics due to an increase in the number of households. (**a**) Number of households *N*(*t*) vs. *t*. (**b**) Mean price of occupied dwellings vs. *t* at various distances *r* (see legend) from the peak of *U*_0_, where gray lines represent initiation and cessation of household creation. (**c**) Mean price of occupied dwellings vs. *r* at various *t* (see legend). (**d**) Spatial density of households *d*(*r*) vs. *r* at various *t* (see legend).

We now provide an example to demonstrate that the model dynamics incorporate the relationship between supply and demand. We initialize a simulation using the same parameters as in Fig 8 in Sec. 8.2 with general dwelling utility *U*_0_ peaking at *U*_0max_ = $5 × 10^3^ y^−1^ at the center of the simulation and **s**(p), **q**(p) and **B**(r) restricted to zeroth elements only. We allow the system to evolve for 40 years, at which point it is close to a steady state. Then we increase the housing demand by increasing the number of households *N* from its initial value of 1000 by 3% per year until it reaches the number of dwellings *M* = 3000 at *t* ≈ 77 years, after which there is no further increase. New households are randomly assigned a wealth level from the same distribution as the original cohort. The resulting evolution is shown in Fig 14(a).

During the initial approach to steady state, Fig 14(b) shows that dwelling prices within the central regions rise steeply and rapidly stabilize as wealthy households move to near the peak of *U*_0_. This effect propagates outward, with a gradual fall in prices in the countryside as population migrates toward the center. By *t* = 20 y this process is almost complete, and there is little further change prior to *t* = 40 y. Fig 14(c) shows that this leads to a spatial price profile that changes only slightly between 20 and 40 years into the simulation, as was seen in Fig 10(a). Fig 14(d) shows that the density of households rises from its initial value to saturation in the core of the simulation, while the countryside depopulates.

Once the population begins to rise from *t* = 40 years onward Fig 14(b) shows that prices start to rise at large *r* because the increased number of wealthy households require more space near the center, thereby forcing others outward and the boundaries of the city to expand. The increasing density of households on the fringes forces prices upward due to the contribution of *q*_0_ to dwelling prices. This does not occur in inner regions where the density is close to maximal already. (As noted previously, in the real world, investment and resulting construction here would push densities and prices upward near *r* = 0, but we do not explore these effects here.) As the number of households *N* approaches the upper limit of the number of dwellings *M*, dwelling prices in the outer regions begin to plateau as the density becomes uniform, as seen in Fig 14(d), and only the *U*_0_ term changes with **r**. After *t* = 77 y a steady state is reached and no significant further net evolution occurs.

Further directions to explore would be to allow for migration in and out of the overall system, with simultaneous construction and demolition, as in the schematic in Fig 6. As a proxy for investment-related decisions, one could initiate construction in areas where dwellings are all occupied and demolition of long-unoccupied dwellings (the latter was done in some of the examples in earlier sections). More generally, expected profit from realizing interaction utility in high-density areas could be factored into an investment model, which would also need to take into account risk, interest rates, alternative investment possibilities, and similar considerations.

## 9 Summary and discussion

We have developed a unified mechanistic model of the dynamics and evolution of urban structure and population using just a few simple components to draw together the relevant aspects of housing preference, income, utility, and the effects of supply and demand. We have illustrated the model’s key features and predictions via a series of proof-of-principle applications that yield realistic emergent outcomes for urban structure and dynamics, and have suggested further uses and directions for generalization. The key features of the present work are:

- The core of the model is an approximation to household utility that includes a general contribution that applies to all households and arises from factors such as geography and planning constraints. A second contribution results from a vector of household preferences for particular dwelling characteristics and price, with the latter constrained by household income. A corresponding vector of dwelling characteristics includes both intrinsic features and a land-value contribution inferred from neighborhood dwelling values. A third contribution is expressed via a vector of preferences for characteristics of neighbors and includes a general interaction utility that expresses the contribution to utility that arises from concentration of population. The resulting utility is similar in structure to that of a high dimensional spin system in an external high dimensional vector field.
- Prices of dwellings are determined by the utility of those whose utility would be increased by acquiring them and a market is continually driven by changes in the numbers of households and dwellings and movements of households on- and off-market. Transactions occur as a result, leading to market evolution and spatial migration of households over time.
- Simulations show that initially uniform distributions of population evolve into concentrated clusters that qualitatively resemble real polycentric urban concentrations. This occurs even without imposing any preferred locations because the interaction utility favors higher population density.
- Population concentrations also form around peaks of general utility (e.g., transport or employment hubs) with income stratification evolving over time: wealthier households concentrate toward the peak, while poorer ones migrate to the fringes where housing is cheaper. In the countryside, housing costs fall as population density decreases, whereas they rise in the central regions. All these features are consistent with what is seen in real urban areas.
- Similar effects to (iv) are seen when a particular favored dwelling feature is geographically concentrated (e.g., lakeside views). In this case, households with a preference for that feature concentrate near its peak, whereas others form clusters elsewhere, as seen in actual cities.
- Aside from its effect in promoting high population densities, terms in the interaction utility can lead to segregation of mutually hostile groups, leading to nearly homogeneous clusters or neighborhoods separated by sharp boundaries. If the strength and/or range of this term are sufficiently strong, this can reduce the utility of both groups by forcing them away from the peak of general utility. This sets a financial scale for economic countermeasures that would be required to prevent or reverse such segregation.
- An increase of housing demand over supply was shown to result in price rises and an expansion of the central city core in which population density is maximal, forcing poorer households further out. These effects are commonly seen in real cities, driving urban sprawl and socioeconomic stratification.

For the first time, the above results bring together many aspects of urban structure and population dynamics that have previously been considered separately. By interrelating multiple effects on a single utility scale in terms of monetary value, they can potentially assist in guiding financial and policy interventions to improve urban structure.

Many features remain to be included, such as resulting demands on transport and other infrastructure, how private and public housing investment decisions are driven by public utility and profit to investors, and how policy could be used to influence structure via income support and changes in general utility. Similarly, many further applications that immediately present themselves have not been explored either individually or in combination, although some have been mentioned in preceding sections. Notable amongst these would to incorporate the coupled evolution of transport capacity and population distribution, including detailed geographical constraints on transport routes. Likewise, coevolution of employment and population could be modeled by viewing employers as a special type of “household” that has a preference for high urban densities (or low ones for farming, for example) and particular household characteristics (e.g., educational level).

Statistics of cluster size and its relationship to critical dynamics and Zipf’s law could also be explored via larger simulations or multiple realizations of city formation starting from different initial conditions. Going beyond housing, one might also apply the model to treat other goods that are geographically immobile and to fields such as opinion dynamics, in which individuals cluster into political parties, social clubs, internet echo chambers, and religions. In the natural world, there may also be application to formation of precursor populations of new species via combined spatial segregation and sexual selection.

## Supporting information

S1 Video Polycentric city formation. Time evolution from random initial conditions to the polycentric structure seen in Fig 7(g).
(GIF)

S2 Video Segregation within and between cities. Time evolution from random initial conditions to the polycentric segregated structure seen in Fig 13.
(GIF)
