# Supplementary figures and images for: Spatiotemporal evolution of urban populations and housing: A dynamic utility-driven market-mediated model
Source: PLoS One. 2023 Apr 7;18(4):e0282583. doi: 10.1371/journal.pone.0282583 (PMC10081807; doi:10.1371/journal.pone.0282583)

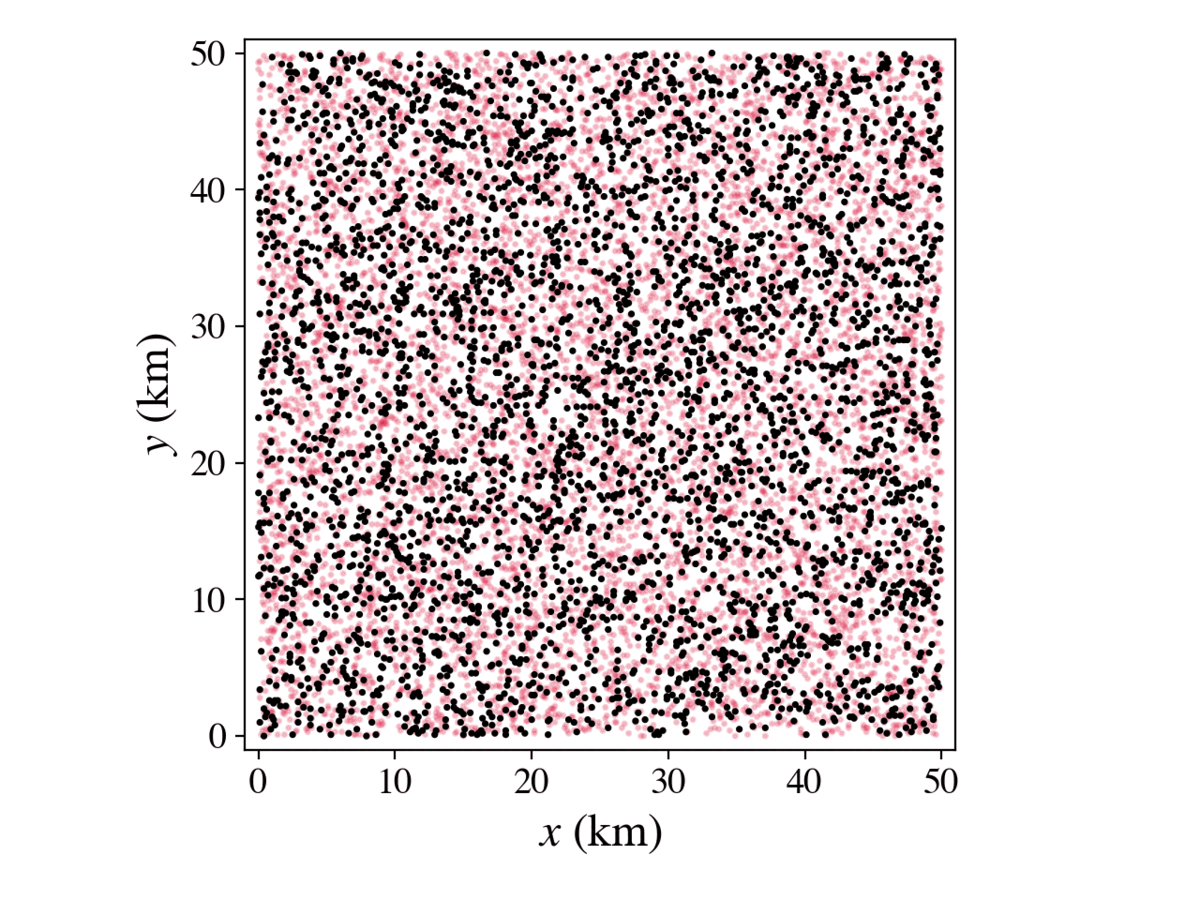

Supplement: S1 Video — Time evolution from random initial conditions to the polycentric structure seen in Fig 7(g). (GIF) [file pone.0282583.s001.gif]

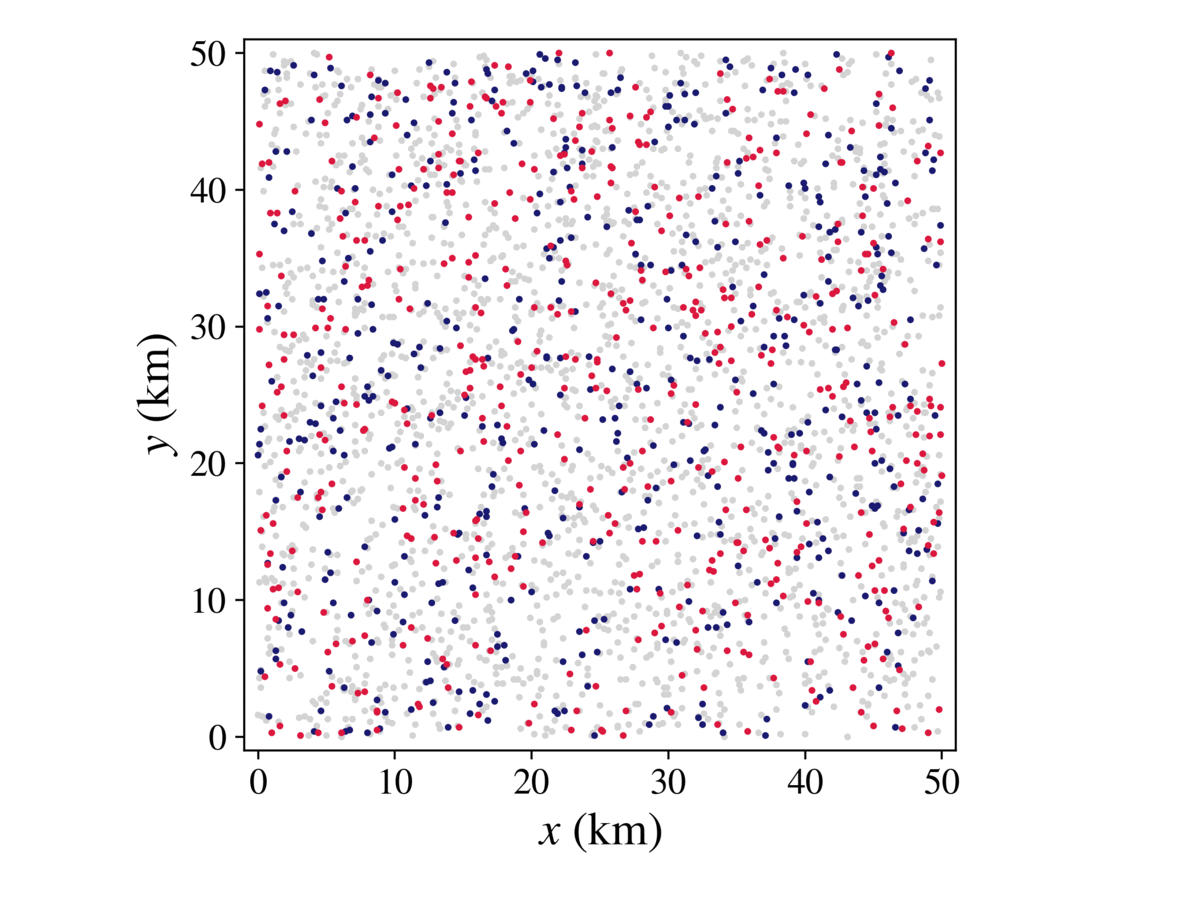

Supplement: S2 Video — Time evolution from random initial conditions to the polycentric segregated structure seen in Fig 13. (GIF) [file pone.0282583.s002.gif]
